# Supplementary material for: Derepression of the epithelial transcription factor GRHL2 promotes direct hepatocyte-to-cholangiocyte transdifferentiation
Source: PLoS Biol. 2025 Dec 12;23(12):e3003547. doi: 10.1371/journal.pbio.3003547 (PMC12714216; doi:10.1371/journal.pbio.3003547)
Supplement: S1 Fig — (PDF) [file pbio.3003547.s001.pdf]

Fig.S1

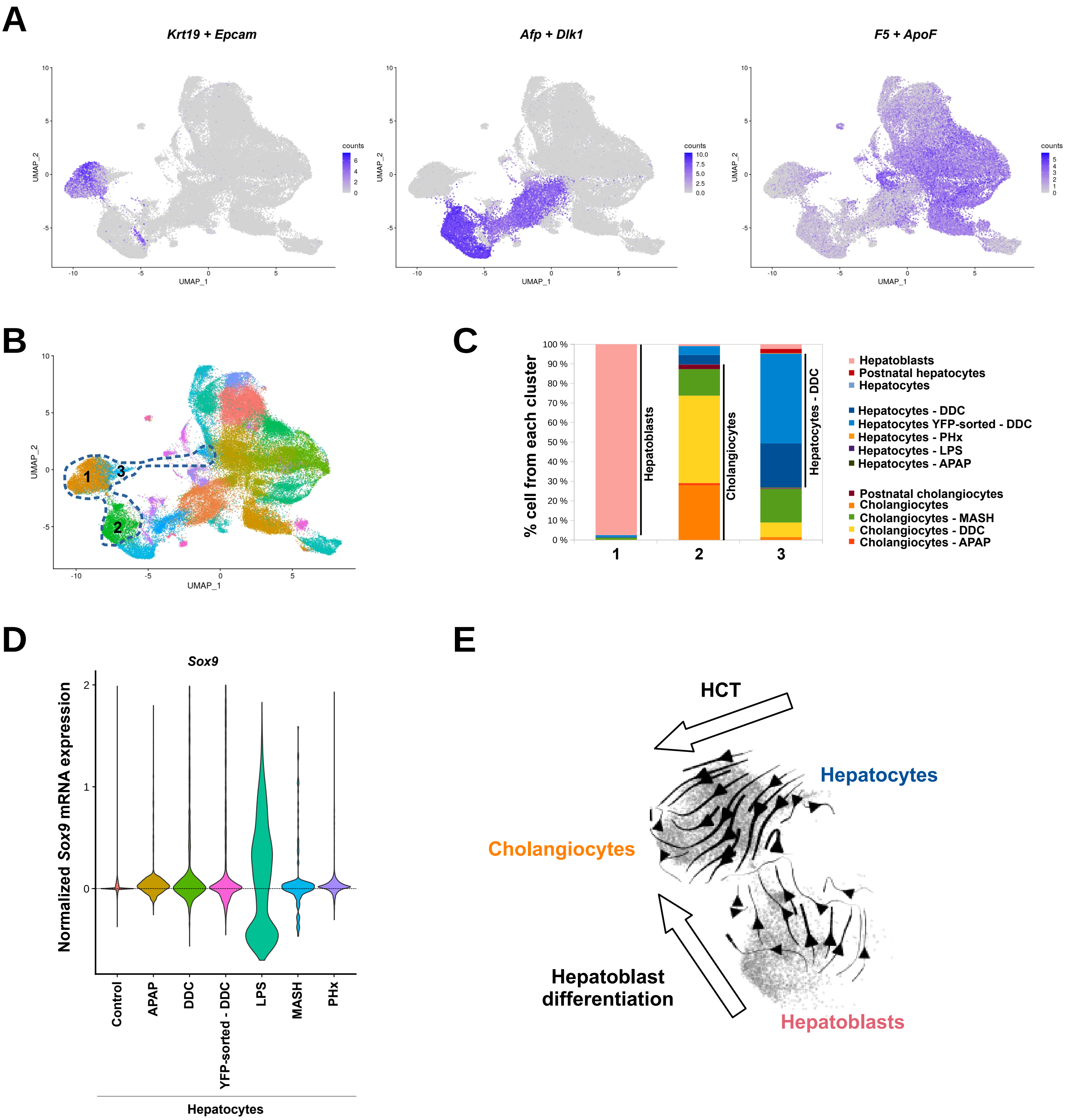
A
Krt19 + Epcam
Afp + Dlk1
F5 + ApoF
counts
UMAP_1
UMAP_2
B
C
% cell from each cluster
Hepatoblasts
Cholangiocytes
Hepatocytes - DDC
Hepatoblasts
Postnatal hepatocytes
Hepatocytes
Hepatocytes - DDC
Hepatocytes YFP-sorted - DDC
Hepatocytes - PHx
Hepatocytes - LPS
Hepatocytes - APAP
Postnatal cholangiocytes
Cholangiocytes
Cholangiocytes - MASH
Cholangiocytes - DDC
Cholangiocytes - APAP
D
Sox9
Normalized Sox9 mRNA expression
Control
APAP
DDC
YFP-sorted - DDC
LPS
MASH
PHx
Hepatocytes
E
HCT
Hepatocytes
Cholangiocytes
Hepatoblast differentiation
Hepatoblasts

Fig.S1

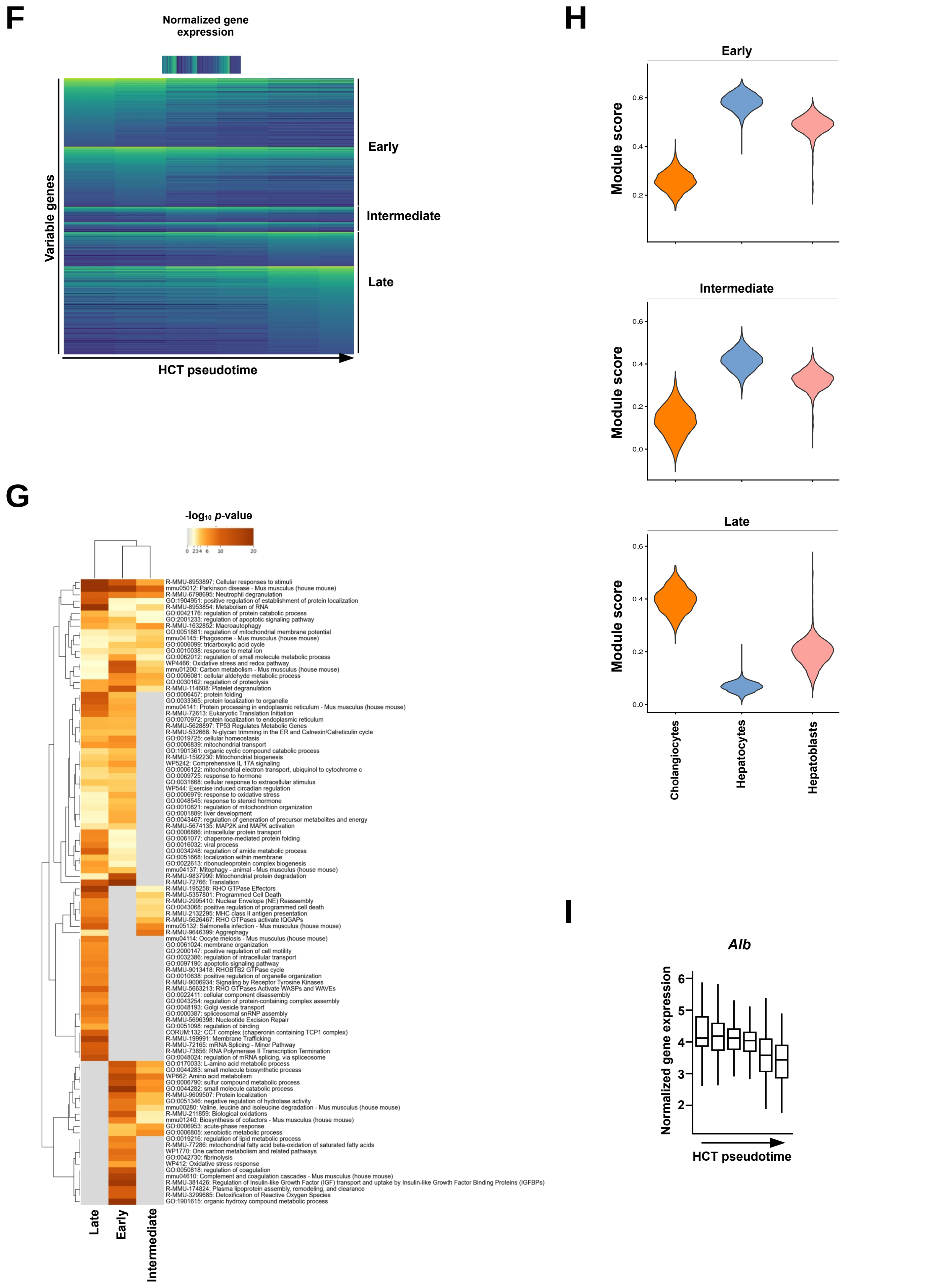
F
Normalized gene expression
Variable genes
Early
Intermediate
Late
HCT pseudotime
G
-log10 p-value
0 2 3 4 6 10 20
R-MMU-8953897: Cellular responses to stimuli
mmu05012: Parkinson disease - Mus musculus (house mouse)
R-MMU-6798695: Neutrophil degranulation
GO:1904951: positive regulation of establishment of protein localization
R-MMU-8953854: Metabolism of RNA
GO:0042176: regulation of protein catabolic process
GO:2001233: regulation of apoptotic signaling pathway
R-MMU-1632852: Macroautophagy
GO:0051881: regulation of mitochondrial membrane potential
mmu04145: Phagosome - Mus musculus (house mouse)
GO:0006099: tricarboxylic acid cycle
GO:0010038: response to metal ion
GO:0062012: regulation of small molecule metabolic process
WP4466: Oxidative stress and redox pathway
mmu01200: Carbon metabolism - Mus musculus (house mouse)
GO:0006081: cellular aldehyde metabolic process
GO:0030162: regulation of proteolysis
R-MMU-114608: Platelet degranulation
GO:0006457: protein folding
GO:0033365: protein localization to organelle
mmu04141: Protein processing in endoplasmic reticulum - Mus musculus (house mouse)
R-MMU-72613: Eukaryotic Translation Initiation
GO:0070972: protein localization to endoplasmic reticulum
R-MMU-5628897: TP53 Regulates Metabolic Genes
R-MMU-532668: N-glycan trimming in the ER and Calnexin/Calreticulin cycle
GO:0019725: cellular homeostasis
GO:0006839: mitochondrial transport
GO:1901361: organic cyclic compound catabolic process
R-MMU-1592230: Mitochondrial biogenesis
WP5242: Comprehensive IL 17A signaling
GO:0006122: mitochondrial electron transport, ubiquinol to cytochrome c
GO:0009725: response to hormone
GO:0031668: cellular response to extracellular stimulus
WP544: Exercise induced circadian regulation
GO:0006979: response to oxidative stress
GO:0048545: response to steroid hormone
GO:0010821: regulation of mitochondrion organization
GO:0001889: liver development
GO:0043467: regulation of generation of precursor metabolites and energy
R-MMU-5674135: MAP2K and MAPK activation
GO:0006886: intracellular protein transport
GO:0061077: chaperone-mediated protein folding
GO:0016032: viral process
GO:0034248: regulation of amide metabolic process
GO:0051668: localization within membrane
GO:0022613: ribonucleoprotein complex biogenesis
mmu04137: Mitophagy - animal - Mus musculus (house mouse)
R-MMU-9837999: Mitochondrial protein degradation
R-MMU-72766: Translation
R-MMU-195258: RHO GTPase Effectors
R-MMU-5357801: Programmed Cell Death
R-MMU-2995410: Nuclear Envelope (NE) Reassembly
GO:0043068: positive regulation of programmed cell death
R-MMU-2132295: MHC class II antigen presentation
R-MMU-5626467: RHO GTPases activate IQGAPs
mmu05132: Salmonella infection - Mus musculus (house mouse)
R-MMU-9646399: Aggrephagy
mmu04114: Oocyte meiosis - Mus musculus (house mouse)
GO:0061024: membrane organization
GO:2000147: positive regulation of cell motility
GO:0032386: regulation of intracellular transport
GO:0097190: apoptotic signaling pathway
R-MMU-9013418: RHOBTB2 GTPase cycle
GO:0010638: positive regulation of organelle organization
R-MMU-9006934: Signaling by Receptor Tyrosine Kinases
R-MMU-5663213: RHO GTPases Activate WASPs and WAVEs
GO:0022411: cellular component disassembly
GO:0043254: regulation of protein-containing complex assembly
GO:0048193: Golgi vesicle transport
GO:0000387: spliceosomal snRNP assembly
R-MMU-5696398: Nucleotide Excision Repair
GO:0051098: regulation of binding
CORUM:132: CCT complex (chaperonin containing TCP1 complex)
R-MMU-199991: Membrane Trafficking
R-MMU-72165: mRNA Splicing - Minor Pathway
R-MMU-73856: RNA Polymerase II Transcription Termination
GO:0048024: regulation of mRNA splicing, via spliceosome
GO:0170033: L-amino acid metabolic process
GO:0044283: small molecule biosynthetic process
WP662: Amino acid metabolism
GO:0006790: sulfur compound metabolic process
GO:0044282: small molecule catabolic process
R-MMU-9609507: Protein localization
GO:0051346: negative regulation of hydrolase activity
mmu00280: Valine, leucine and isoleucine degradation - Mus musculus (house mouse)
R-MMU-211859: Biological oxidations
mmu01240: Biosynthesis of cofactors - Mus musculus (house mouse)
GO:0006953: acute-phase response
GO:0006805: xenobiotic metabolic process
GO:0019216: regulation of lipid metabolic process
R-MMU-77286: mitochondrial fatty acid beta-oxidation of saturated fatty acids
WP1770: One carbon metabolism and related pathways
GO:0042730: fibrinolysis
WP412: Oxidative stress response
GO:0050818: regulation of coagulation
mmu04610: Complement and coagulation cascades - Mus musculus (house mouse)
R-MMU-381426: Regulation of Insulin-like Growth Factor (IGF) transport and uptake by Insulin-like Growth Factor Binding Proteins (IGFBPs)
R-MMU-174824: Plasma lipoprotein assembly, remodeling, and clearance
R-MMU-3299685: Detoxification of Reactive Oxygen Species
GO:1901615: organic hydroxy compound metabolic process
Late
Early
Intermediate
H
Early
Module score
0.2
0.4
0.6
Intermediate
Module score
0.0
0.2
0.4
0.6
Late
Module score
0.0
0.2
0.4
0.6
Cholangiocytes
Hepatocytes
Hepatoblasts
I
Alb
Normalized gene expression
2
3
4
5
6
HCT pseudotime

Supplementary Fig.1: Additional characterization of the mouse parenchymal liver cells scRNA-seq atlas and of the cellular trajectory involved in HCT

(A) UMAP from Fig.1A was used to project expression of the indicated markers genes: *Afp* and *Dlk1* for hepatoblasts, *F5* and *ApoF* for hepatocytes and *Krt19* and *Epcam* for cholangiocytes. Cumulative expression of the 2 marker genes was used.

(B) UMAP from Fig.1A was used to display the result of cell clustering and the 3 selected clusters from Fig.1B were further highlighted.

(C) Dataset of origin of hepatocytes from clusters 1-3. Horizontal bars were added to highlight the main cell type in each one of the 3 clusters.

(D) Violin plots showing the normalized mRNA expression levels of *Sox9* in the indicated subsets of mature hepatocytes.

(E) An area from Fig.1D centered on cholangiocytes is shown to highlight cell transitions precited by RNA velocity analyses and associated with hepatobalst differentiation or HCT.

(F) Heatmap showing the normalized genes expression of variable genes along the course of HCT pseudotime. Genes were ranked according to the time at which they were the most highly expressed ranging from early to late. This allowed to discriminate 3 groups of genes indicated as early, intermediate and late.

(G) Heatmap showing the results of biological term enrichments analyses performed using Metascape for early, intermediate and late genes identified in panel F. The dendrogram on the left shows the hierarchical clustering based on $-\log_{10}$ *p*-values as provided by Metascape.

(H) Cells used for RNA velocity analyses were subsequently utilized to mine expression of early, intermediate and late genes identified in panel F in hepatoblasts, healthy hepatocytes and cholangiocytes.

(I) Boxplot showing the normalized expression of *Alb* in discrete hepatocyte cell subsets along the course of HCT as defined using pseudotime analysis similarly to Fig.1G.
