# Supplementary material for: Derepression of the epithelial transcription factor GRHL2 promotes direct hepatocyte-to-cholangiocyte transdifferentiation
Source: PLoS Biol. 2025 Dec 12;23(12):e3003547. doi: 10.1371/journal.pbio.3003547 (PMC12714216; doi:10.1371/journal.pbio.3003547)
Supplement: S2 Fig — (PDF) [file pbio.3003547.s002.pdf]

Fig. S2

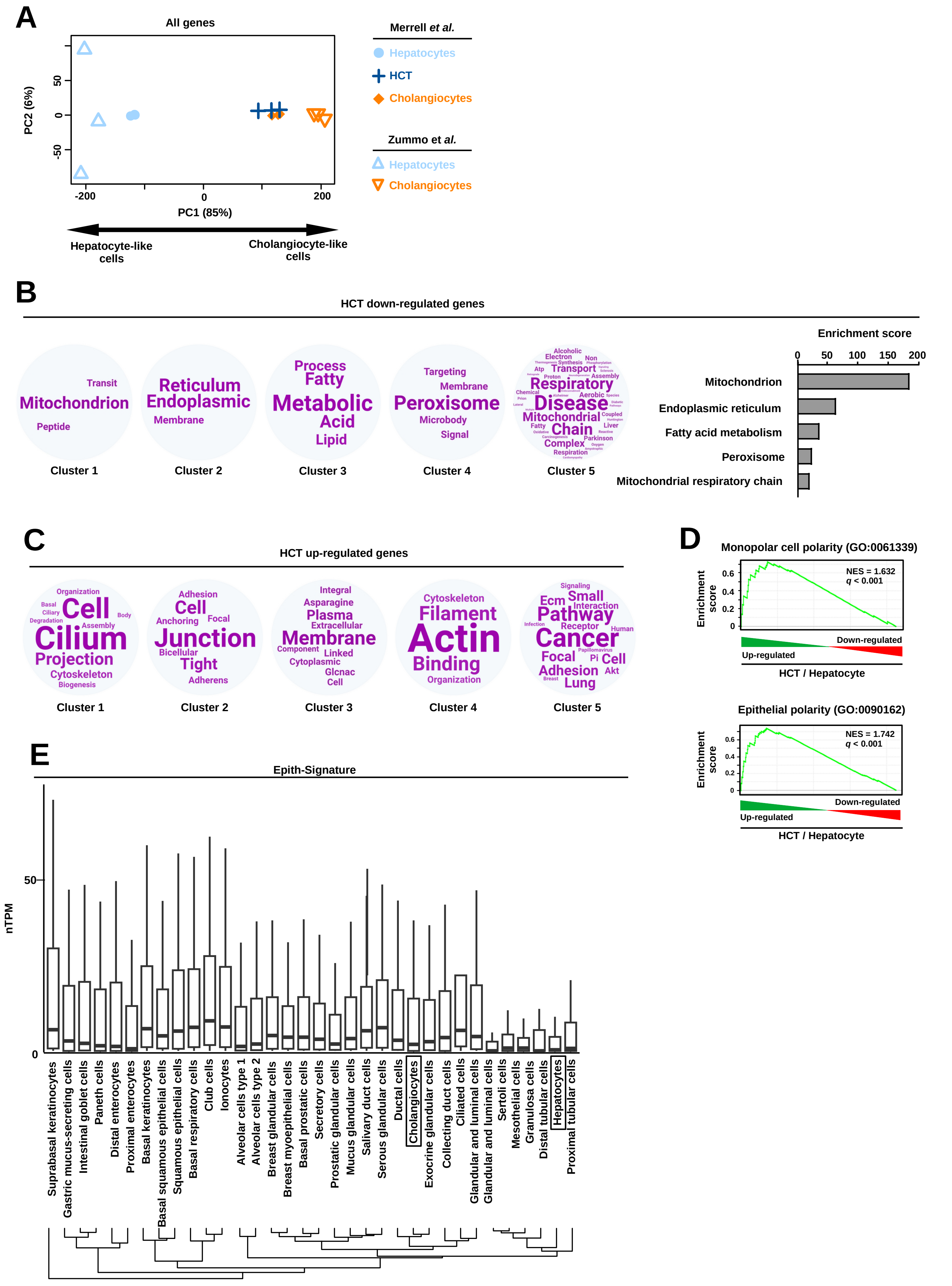
A
All genes
PC2 (6%)
PC1 (85%)
-200
0
200
50
0
-50
Hepatocyte-like cells
Cholangiocyte-like cells
Merrell et al.
Hepatocytes
HCT
Cholangiocytes
Zummo et al.
Hepatocytes
Cholangiocytes
B
HCT down-regulated genes
Transit
Mitochondrion
Peptide
Cluster 1
Reticulum
Endoplasmic
Membrane
Cluster 2
Process
Fatty
Metabolic
Acid
Lipid
Cluster 3
Targeting
Membrane
Peroxisome
Microbody
Signal
Cluster 4
Alcoholic
Electron
Transport
Respiratory
Disease
Mitochondrial
Chain
Complex
Respiration
Cluster 5
Enrichment score
0
50
100
150
200
Mitochondrion
Endoplasmic reticulum
Fatty acid metabolism
Peroxisome
Mitochondrial respiratory chain
C
HCT up-regulated genes
Organization
Cell
Cilium
Projection
Cytoskeleton
Biogenesis
Cluster 1
Adhesion
Cell
Anchoring
Focal
Junction
Bicellular
Tight
Adherens
Cluster 2
Integral
Asparagine
Plasma
Extracellular
Membrane
Component
Linked
Cytoplasmic
Glcnac
Cell
Cluster 3
Cytoskeleton
Filament
Actin
Binding
Organization
Cluster 4
Signaling
Ecm
Small
Pathway
Interaction
Receptor
Cancer
Focal
Cell
Adhesion
Lung
Cluster 5
D
Monopolar cell polarity (GO:0061339)
Enrichment score
NES = 1.632
q < 0.001
Down-regulated
Up-regulated
HCT / Hepatocyte
Epithelial polarity (GO:0090162)
Enrichment score
NES = 1.742
q < 0.001
Down-regulated
Up-regulated
HCT / Hepatocyte
E
Epith-Signature
nTPM
50
0
Suprabasal keratinocytes
Gastric mucus-secreting cells
Intestinal goblet cells
Paneth cells
Distal enterocytes
Proximal enterocytes
Basal keratinocytes
Basal squamous epithelial cells
Squamous epithelial cells
Basal respiratory cells
Club cells
Ionocytes
Alveolar cells type 1
Alveolar cells type 2
Breast glandular cells
Breast myoepithelial cells
Basal prostatic cells
Secretory cells
Prostatic glandular cells
Mucus glandular cells
Salivary duct cells
Serous glandular cells
Ductal cells
Cholangiocytes
Exocrine glandular cells
Collecting duct cells
Ciliated cells
Glandular and luminal cells
Glandular and luminal cells
Sertoli cells
Mesothelial cells
Granulosa cells
Distal tubular cells
Hepatocytes
Proximal tubular cells

Supplementary Fig.2: Additional characterization of the gene transcriptional changes occurring in HCT

(A) Visualization of all RNA-seq datasets used in the PCA displayed in Fig.2A. In addition to data from (Merrell et al. 2021) shown in Fig.2A, the healthy hepatocytes and cholangiocytes data from (Zummo et al. 2023) used to initially perform the PCA (see Materials and Methods) are also shown here.

(B) Functional annotation of genes down-regulated during HCT performed similarly to Fig.2B. Word clouds show the results of word counting performed using the name of individual enriched terms in each of the 5 top clusters identified by the DAVID tool. The size of each word is proportional to its number of occurrences. These analyses were used to name these 5 clusters, whose enrichment scores (defined as the best enrichment score among individual terms within each cluster) are shown using a bar graph (right).

(C) Word clouds showing the results of word counting performed using the name of individual enriched terms in each of the 5 top clusters identified by the DAVID tool when mining HCT-up-regulated genes. The size of each word is proportional to its number of occurrences. These analyses were used to name the 5 clusters in Fig.2B.

(D) Enrichment plots obtained using GSEA performed with the gene ontology terms "Establishment or maintenance of monopolar cell polarity" (GO:0061339) or "Establishment of epithelial cell polarity" (GO:0090162) as the gene sets and transcriptomic changes induced by HCT defined as in Fig.2B from (Merrell et al. 2021). NES stands for normalized enrichment score.

(E) Box plot showing the expression of genes from the Epith-Signature (Breschi et al. 2020) in each indicated individual epithelial cell type. The dendrogram at the bottom shows the results of hierarchical clustering analysis performed on these data. Cholangiocytes and hepatocytes are highlighted using frames. nTPM stands for normalized transcripts per million.
