# Supplementary material for: Derepression of the epithelial transcription factor GRHL2 promotes direct hepatocyte-to-cholangiocyte transdifferentiation
Source: PLoS Biol. 2025 Dec 12;23(12):e3003547. doi: 10.1371/journal.pbio.3003547 (PMC12714216; doi:10.1371/journal.pbio.3003547)
Supplement: S3 Fig — (PDF) [file pbio.3003547.s003.pdf]

Fig.S3

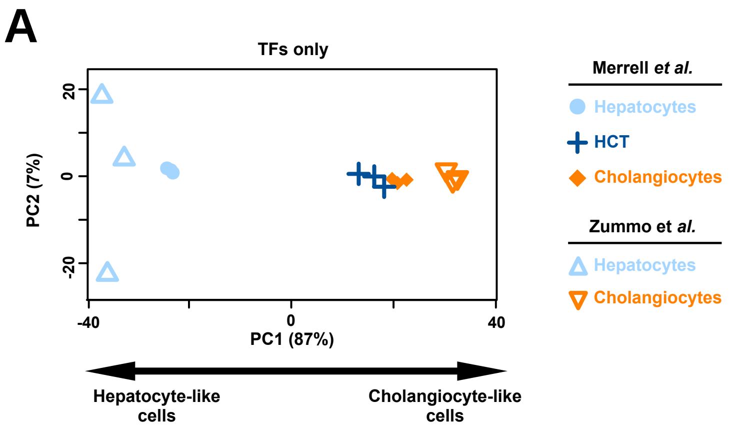
A
TFs only
PC2 (7%)
20
0
-20
-40
0
40
PC1 (87%)
Hepatocyte-like cells
Cholangiocyte-like cells
Merrell et al.
Hepatocytes
HCT
Cholangiocytes
Zummo et al.
Hepatocytes
Cholangiocytes

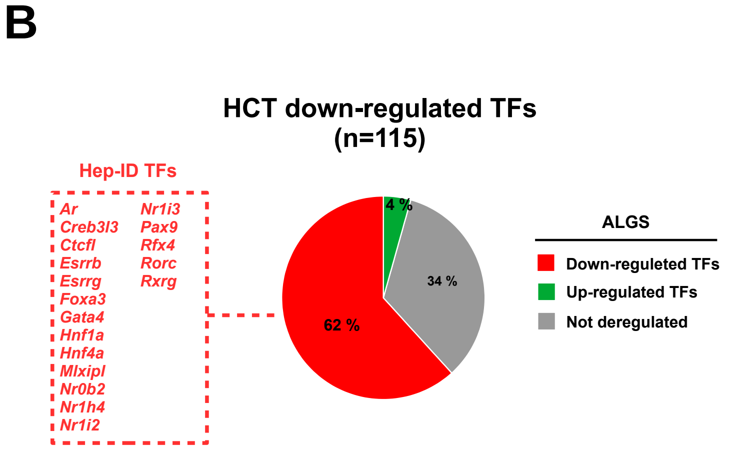
B
HCT down-regulated TFs (n=115)
Hep-ID TFs
Ar
Creb3l3
Ctcfl
Esrrb
Esrrg
Foxa3
Gata4
Hnf1a
Hnf4a
Mlxipl
Nr0b2
Nr1h4
Nr1i2
Nr1i3
Pax9
Rfx4
Rorc
Rxrg
4 %
34 %
62 %
ALGS
Down-regueted TFs
Up-regulated TFs
Not deregulated

Supplementary Fig.3: Additional characterization of deregulated TF-encoding genes during HCT

(A) Visualization of all RNA-seq datasets used in the PCA displayed in Fig.3A. In addition to data from (Merrell et al. 2021) shown in Fig.3A, the healthy hepatocytes and cholangiocytes data from (Zummo et al. 2023) used to initially perform the PCA (see Materials and Methods) are also shown here.

(B) TF-encoding genes down-regulated during HCT were obtained by comparing bulk RNA-seq data from lineage-traced hepatocytes undergoing HCT from DDC-treated mice compared to that of control hepatocytes from healthy livers from (Merrell et al. 2021) (q-value < 0.05 and $\log_2$ FC < 0). The obtained gene list was monitored for changes in hepatocytes undergoing HCT in another mouse model, i.e. model of ALGS lacking an intrahepatic biliary system at birth (Schaub et al. 2018). In this second dataset, genes with q-value < 0.15 and $\log_2$ FC < 0 or > 0 were defined as down- or up-regulated, respectively. Genes with q-value > 0.15 were considered as “Not deregulated”. Hepatocyte identity (Hep-ID) TFs, as defined in our previous study (Dubois-Chevalier et al. 2023), which were down-regulated both in DDC- and ALGS-related HCT are indicated in red.
