# Supplementary material for: Derepression of the epithelial transcription factor GRHL2 promotes direct hepatocyte-to-cholangiocyte transdifferentiation
Source: PLoS Biol. 2025 Dec 12;23(12):e3003547. doi: 10.1371/journal.pbio.3003547 (PMC12714216; doi:10.1371/journal.pbio.3003547)
Supplement: S4 Fig — (PDF) [file pbio.3003547.s004.pdf]

Fig. S4

A

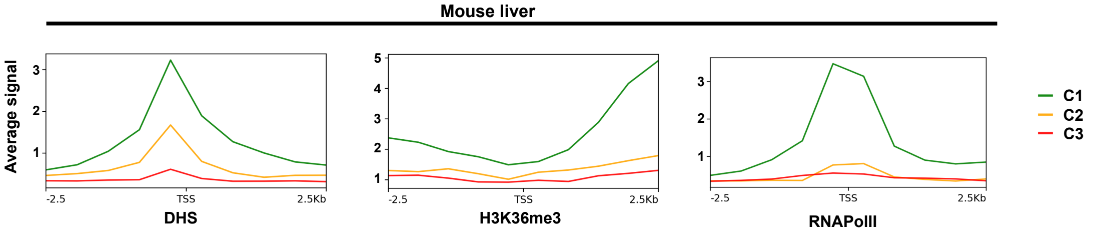
Mouse liver
Average signal
DHS
H3K36me3
RNAPolII
-2.5
TSS
2.5Kb
C1
C2
C3

B

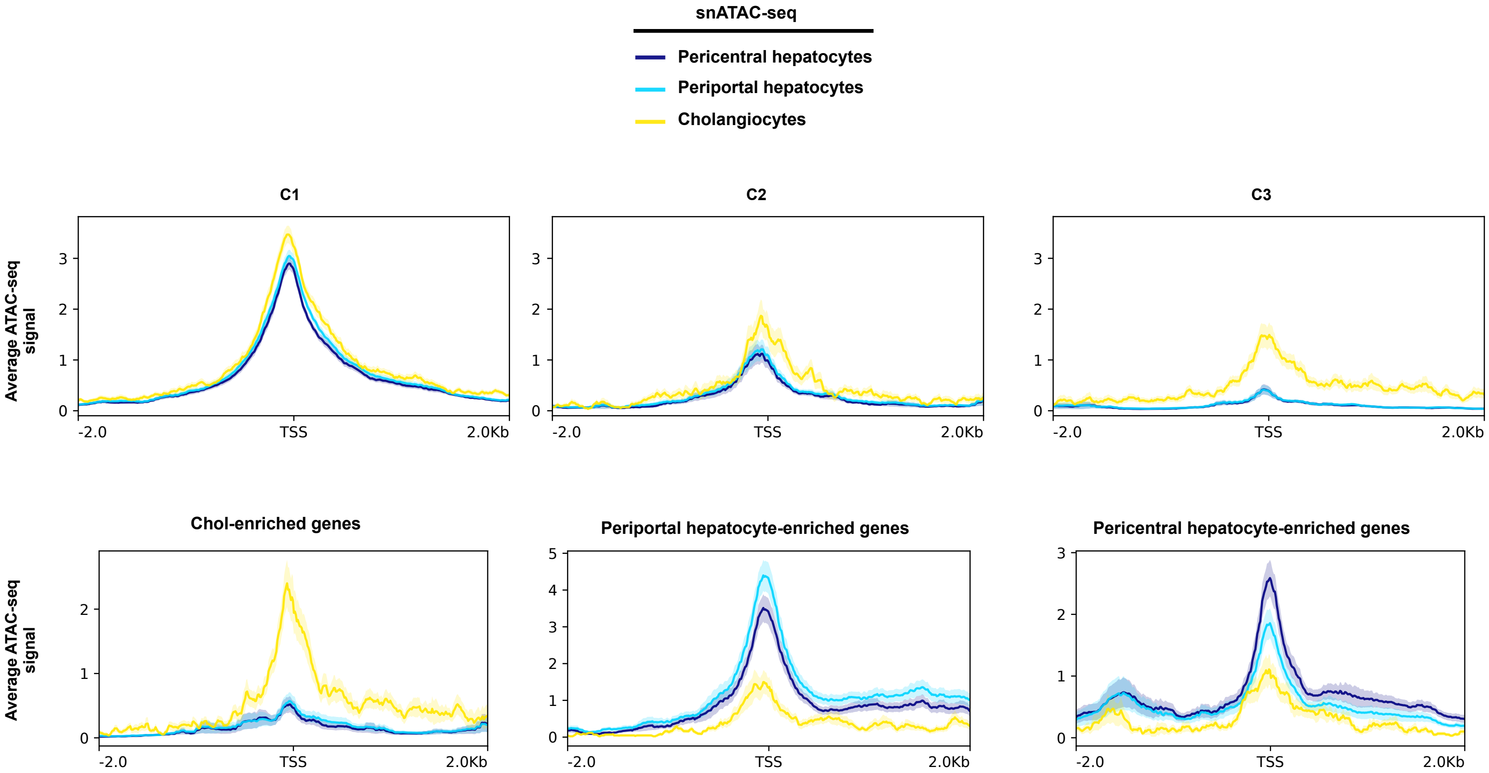
snATAC-seq
Pericentral hepatocytes
Periportal hepatocytes
Cholangiocytes
C1
C2
C3
Average ATAC-seq signal
-2.0
TSS
2.0Kb
Chol-enriched genes
Periportal hepatocyte-enriched genes
Pericentral hepatocyte-enriched genes

Supplementary Fig.4: Average DHS-seq, H3K36me3 and RNAPolII ChIP-seq signals at the promoters of genes from clusters C1-3 in the mouse liver

(A) Average DHS-seq or ChIP-Seq signals for H3K36me3 or RNA polymerase II (RNAPolII) at promoters from clusters C1-3 (defined in Fig.3D) in the mouse liver. Low signal was arbitrarily set to 1. Signals are shown in windows of +/- 2.5 kb around the genes TSS.

(B) Average ATAC-seq signals at promoters from clusters C1-3 (defined in Fig.3D; top) or at promoters of genes whose expression is enriched in cholangiocytes, periportal or pericentral hepatocytes (bottom) (Supplementary Tables 3 and 4). ATAC-seq signal at individual genes was obtained by aggregating single-nucleus ATAC-seq data from the mouse liver for periportal, pericentral and cholangiocytes (Supplementary Table 4) (Bravo Gonzalez-Blas et al. 2024). Average signals +/-SE found at the indicated gene promoters are shown in windows of +/- 2 kb around the genes TSS.
