# Supplementary material for: Derepression of the epithelial transcription factor GRHL2 promotes direct hepatocyte-to-cholangiocyte transdifferentiation
Source: PLoS Biol. 2025 Dec 12;23(12):e3003547. doi: 10.1371/journal.pbio.3003547 (PMC12714216; doi:10.1371/journal.pbio.3003547)
Supplement: S5 Fig — (PDF) [file pbio.3003547.s005.pdf]

Fig. S5

A

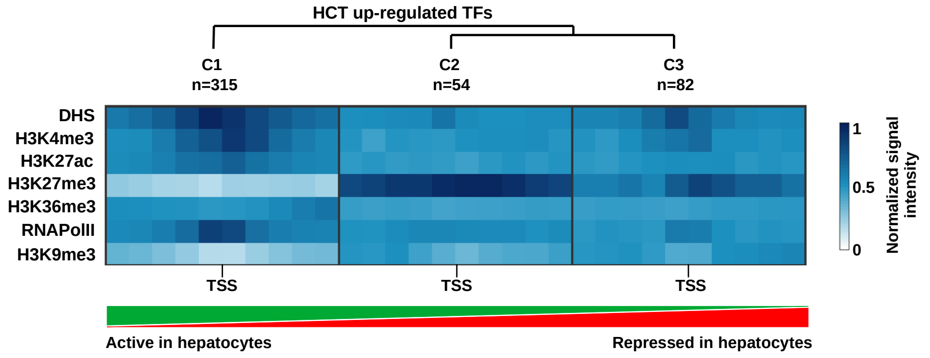
HCT up-regulated TFs
C1
n=315
C2
n=54
C3
n=82
DHS
H3K4me3
H3K27ac
H3K27me3
H3K36me3
RNAPolII
H3K9me3
TSS
TSS
TSS
1
0.5
0
Normalized signal intensity
Active in hepatocytes
Repressed in hepatocytes

B

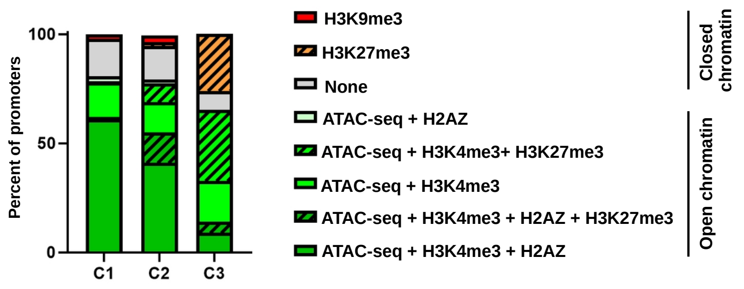
Percent of promoters
100
50
0
C1
C2
C3
H3K9me3
H3K27me3
None
ATAC-seq + H2AZ
ATAC-seq + H3K4me3+ H3K27me3
ATAC-seq + H3K4me3
ATAC-seq + H3K4me3 + H2AZ + H3K27me3
ATAC-seq + H3K4me3 + H2AZ
Closed chromatin
Open chromatin

C

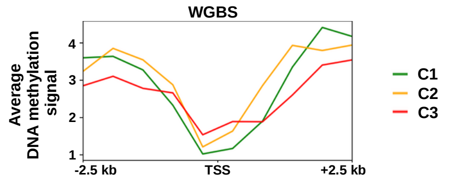
WGBS
Average DNA methylation signal
4
3
2
1
-2.5 kb
TSS
+2.5 kb
C1
C2
C3

D

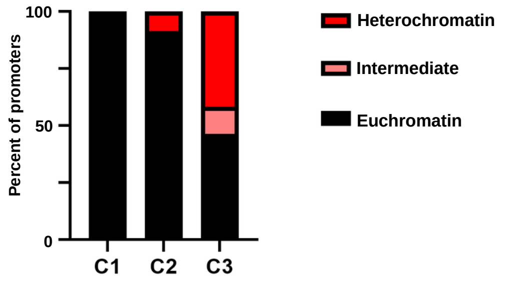
Percent of promoters
100
50
0
C1
C2
C3
Heterochromatin
Intermediate
Euchromatin

Supplementary Fig.5: Promoters from C3 are found in both euchromatin and heterochromatin domains mostly independently of labelling with H3K9me3

(A) Spark-based clustering of promoters from C3 performed as in Fig.3D except H3K9me3 ChIP-seq data from the mouse liver were also used. Average signal for chromatin accessibility (DHS) and indicated modified histones or RNA polymerase II (RNApolII) at TF-encoding gene promoters for the main 3 clusters identified by Spark is shown. The arrows indicate the position of the transcriptional start site (TSS) at the center of the 5 kb windows used for these analyses. Overall, the 3 main clusters obtained are consistent with those reported in Fig.3D indicating H3K9me3 ChIP-seq signal is not a main driver of promoter heterogeneity.

(B) Distribution of promoters from C1-3 within mouse liver chromatin domains defined using modeling the combinatorial presence or absence of signals for ATAC-seq and H2AZ, H3K4me3, H3K27me3 and H3K9me3 ChIP-seq using ChromHMM (Zhang et al. 2021). Enriched signals in the different chromatin domains are shown in the legend and correspondence with the chromatin state numbering used in the original manuscript is as follows: ATAC-seq + H3K4me3 + H2AZ (S2); ATAC-seq + H3K4me3 + H2AZ + H3K27me3 (S2 with H3K27me3); ATAC-seq + H3K4me3 (S1); ATAC-seq + H3K4me3 + H3K27me3 (S1 with H3K27me3); ATAC-seq + H2AZ (S3); H3K27me3 (S6); H3K9me3 (S5).

(C) Average DNA methylation signal at promoters from clusters C1-3 in the mouse liver. Signal is derived from whole-genome shotgun bisulfite sequencing (WGBS; Supplementary Table 4) and is shown in a window of +/- 2.5 kb around the genes TSS. Overall genes from all clusters display DNA hypomethylation around their TSS.

(D) Individual promoters from C1-3 were monitored for their presence in euchromatin or heterochromatin in the mouse liver as defined in (Grindheim et al. 2019) using sonication-resistant heterochromatin sequencing (srHC-seq). Intermediate domains include regions not called as hetero/euchromatic and hetero/euchromatin double-positive regions. The percentage

of promoters in euchromatin, intermediate domains and heterochromatin is shown for the 3 clusters.
