# Supplementary material for: Derepression of the epithelial transcription factor GRHL2 promotes direct hepatocyte-to-cholangiocyte transdifferentiation
Source: PLoS Biol. 2025 Dec 12;23(12):e3003547. doi: 10.1371/journal.pbio.3003547 (PMC12714216; doi:10.1371/journal.pbio.3003547)
Supplement: S6 Fig — (PDF) [file pbio.3003547.s006.pdf]

Fig. S6

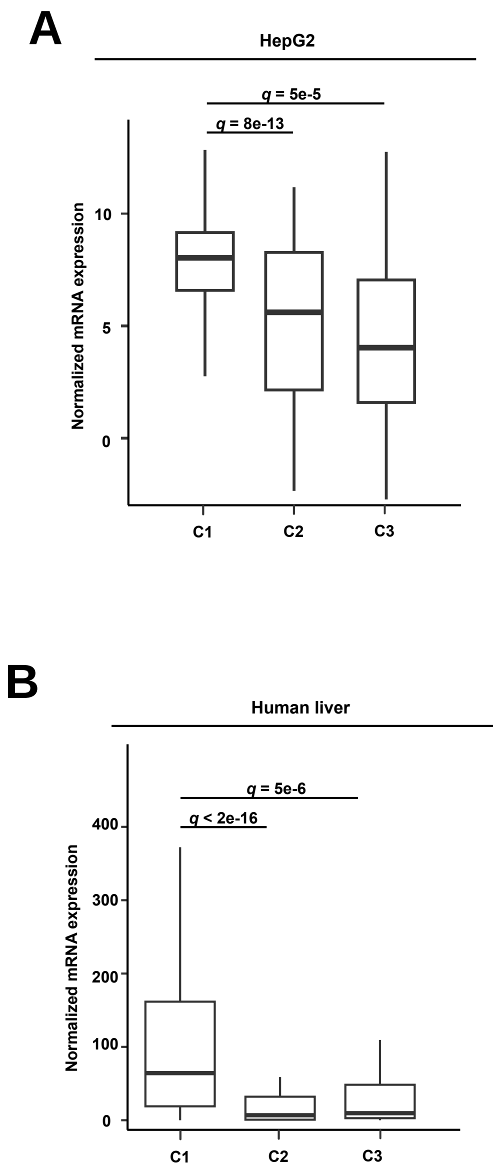
A
HepG2
q = 5e-5
q = 8e-13
Normalized mRNA expression
10
5
0
C1
C2
C3
B
Human liver
q = 5e-6
q < 2e-16
Normalized mRNA expression
400
300
200
100
0
C1
C2
C3

Supplementary Fig.6: Basal expression of TF-encoding genes from the C1-3 clusters

(A) Box plot showing the basal expression of TF-encoding genes from clusters C1-3 in HepG2 cells. Statistical significance was assessed using a Kruskal-Wallis test with Dunn's multiple comparison post hoc test.

(B) Box plot showing the basal expression of TF-encoding genes from clusters C1-3 in the human liver [control samples from our previous RNA-seq analyses (Bou Saleh et al. 2021) were used here]. Statistical significance was assessed using a Kruskal-Wallis test with Dunn's multiple comparison post hoc test.
