# Supplementary material for: Derepression of the epithelial transcription factor GRHL2 promotes direct hepatocyte-to-cholangiocyte transdifferentiation
Source: PLoS Biol. 2025 Dec 12;23(12):e3003547. doi: 10.1371/journal.pbio.3003547 (PMC12714216; doi:10.1371/journal.pbio.3003547)
Supplement: S7 Fig — (PDF) [file pbio.3003547.s007.pdf]

Fig. S7

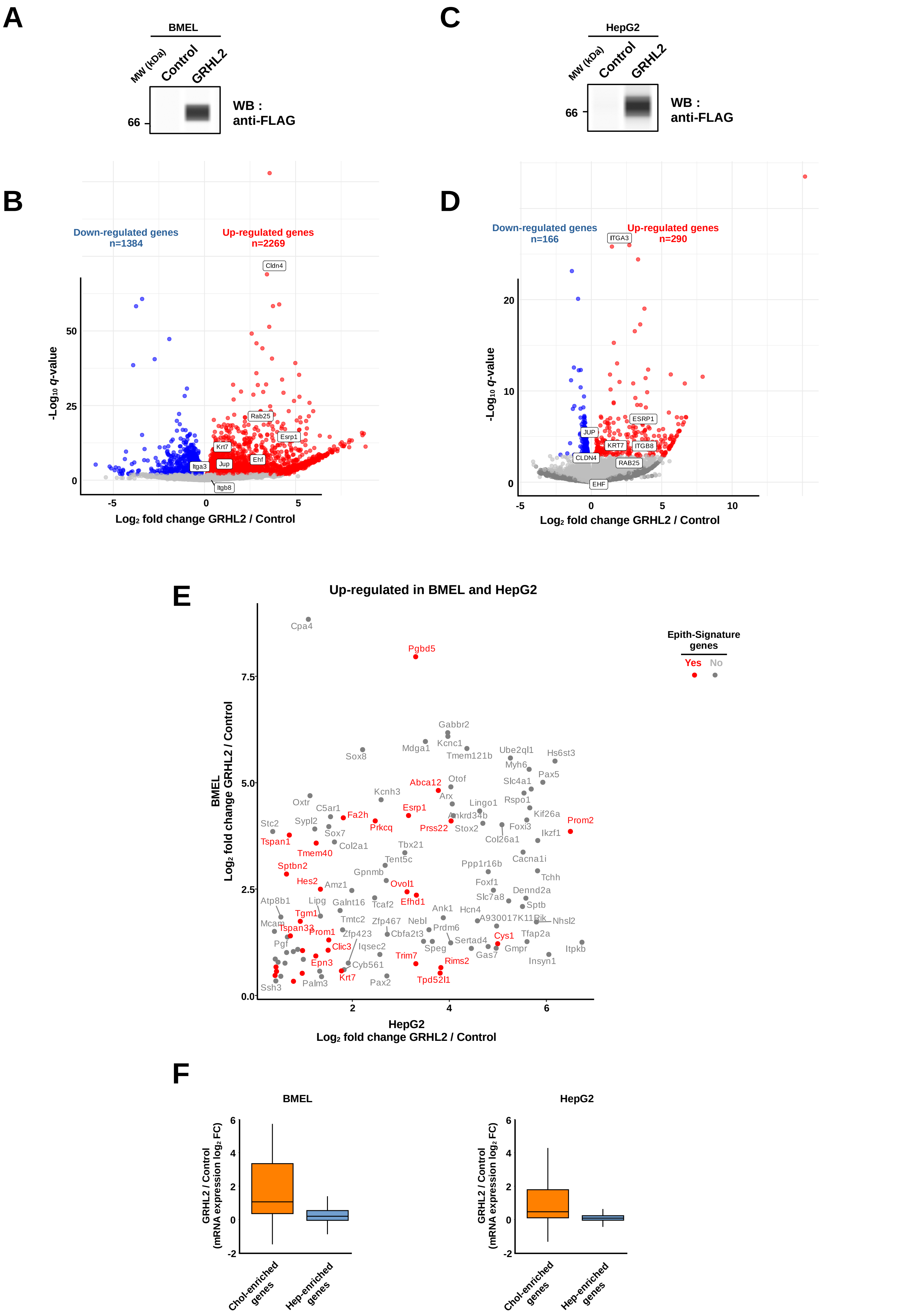
A
BMEL
MW (kDa)
Control
GRHL2
66
WB :
anti-FLAG
C
HepG2
MW (kDa)
Control
GRHL2
66
WB :
anti-FLAG
B
Down-regulated genes
n=1384
Up-regulated genes
n=2269
Cldn4
Rab25
Esrp1
Krt7
Ehf
Itga3
Jup
Itgb8
-Log₁₀ q-value
Log₂ fold change GRHL2 / Control
D
Down-regulated genes
n=166
Up-regulated genes
n=290
ITGA3
ESRP1
JUP
KRT7
ITGB8
CLDN4
RAB25
EHF
-Log₁₀ q-value
Log₂ fold change GRHL2 / Control
E
Up-regulated in BMEL and HepG2
Epith-Signature genes
Yes
No
BMEL
Log₂ fold change GRHL2 / Control
HepG2
Log₂ fold change GRHL2 / Control
Cpa4
Pgbd5
Gabbr2
Kcnc1
Mdga1
Tmem121b
Sox8
Ube2ql1
Hs6st3
Myh6
Pax5
Abca12
Otof
Slc4a1
Kcnh3
Arx
Oxtr
Rspo1
Lingo1
C5ar1
Esrp1
Fa2h
Ankrd34b
Kif26a
Prom2
Sypl2
Prkcq
Prss22
Stox2
Foxi3
Stc2
Sox7
Ikzf1
Tspan1
Col26a1
Col2a1
Tbx21
Tmem40
Tent5c
Cacna1i
Ppp1r16b
Sptbn2
Gpnmb
Tchh
Foxf1
Hes2
Amz1
Ovol1
Dennd2a
Slc7a8
Efhd1
Atp8b1
Lipg
Galnt16
Tcaf2
Sptb
Ank1
Hcn4
Tgm1
A930017K11Rik
Nhsl2
Mcam
Tmtc2
Zfp467
Nebl
Prdm6
Tspan33
Prom1
Zfp423
Cbfa2t3
Tfap2a
Cys1
Pgf
Sertad4
Clic3
Iqsec2
Speg
Gmpr
Itpkb
Trim7
Gas7
Epn3
Cyb561
Rims2
Insyn1
Krt7
Ssh3
Palm3
Pax2
Tpd52l1
F
BMEL
HepG2
GRHL2 / Control
(mRNA expression log₂ FC)
Chol-enriched genes
Hep-enriched genes

Supplementary Fig.7: Additional validation of GRHL2 ectopic expression and characterization of induced transcriptional changes in BMEL and HepG2 cells

(A) Since the construct allowing for GRHL2 ectopic expression included a Flag tag, we performed additional Wes immunoblotting assays on the samples used in Fig.5C with an anti-Flag antibody to monitor GRHL2 levels in transfected BMEL cells. MW, molecular weight. WB, Wes immunoblotting.

(B) Volcano plots showing transcriptomic changes induced by GRHL2 ectopic expression in BMEL cells (RNA-seq data from 4 independent biological replicates used in Fig.5). Significantly regulated genes ($q < 0.05$) are displayed in blue and red for down- and up-regulated genes, respectively.

(C) Since the construct allowing for GRHL2 ectopic expression included a Flag tag, we performed additional Wes immunoblotting assays on the samples used in Fig.6A with an anti-Flag antibody to monitor GRHL2 levels in transfected HepG2 cells. MW, molecular weight. WB, Wes immunoblotting.

(D) Volcano plots showing transcriptomic changes induced by GRHL2 ectopic expression in HepG2 cells (RNA-seq data from 4 independent biological replicates used in Fig.6). Significantly regulated genes ($q < 0.05$) are displayed in blue and red for down- and up-regulated genes, respectively.

(E) Scatter plot of individual genes significantly up-regulated upon GRHL2 ectopic expression in both BMEL and HepG2 cells (defined hereabove) showing their $\log_2$ fold changes in these two models. Additionally, presence of individual genes within the Epith-Signature was highlighted using red dots.

(F) Distribution of the $\log_2$ fold changes for genes of the cholangiocyte-enriched (Chol-enriched) or hepatocyte-enriched (Hep-enriched) lists in BMEL or HepG2 cells ectopically expressing GRHL2.

The original data underlying this figure can be found at the Gene Expression Omnibus (GSE281717).
