# Supplementary material for: Derepression of the epithelial transcription factor GRHL2 promotes direct hepatocyte-to-cholangiocyte transdifferentiation
Source: PLoS Biol. 2025 Dec 12;23(12):e3003547. doi: 10.1371/journal.pbio.3003547 (PMC12714216; doi:10.1371/journal.pbio.3003547)
Supplement: S8 Fig — (PDF) [file pbio.3003547.s008.pdf]

Fig.S8

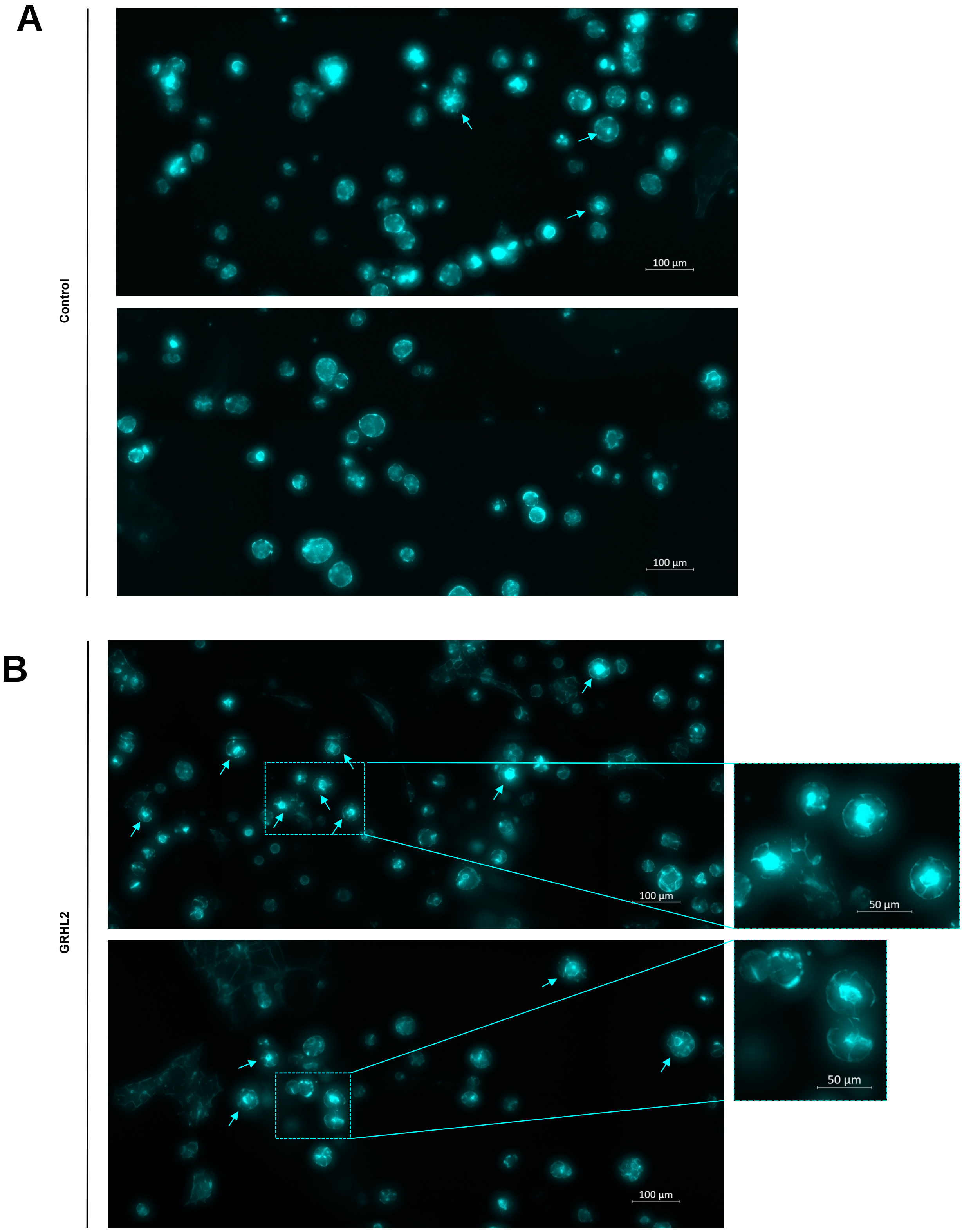

Supplementary Fig.8: Additional representative images of BMEL cell aggregates

BMEL cells were transfected with a GRHL2 expression plasmid or an empty control construct and grown in Matrigel. Additional representative images of obtained aggregates stained with phalloidin are shown. The arrows point to cystic cell aggregates.
