# Supplementary material for: Derepression of the epithelial transcription factor GRHL2 promotes direct hepatocyte-to-cholangiocyte transdifferentiation
Source: PLoS Biol. 2025 Dec 12;23(12):e3003547. doi: 10.1371/journal.pbio.3003547 (PMC12714216; doi:10.1371/journal.pbio.3003547)
Supplement: S9 Fig — (PDF) [file pbio.3003547.s009.pdf]

Fig. S9

A

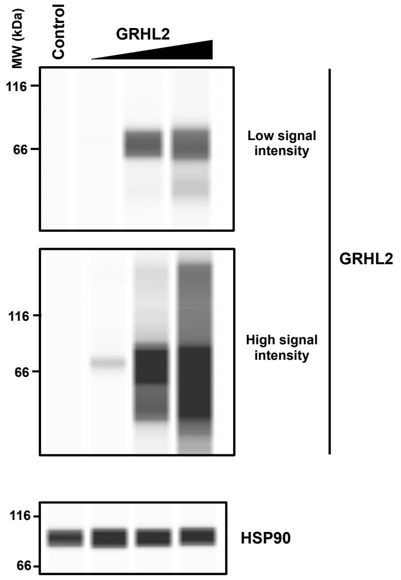

MW (kDa)
Control
GRHL2
116
66
Low signal intensity
116
66
High signal intensity
GRHL2
116
66
HSP90

B

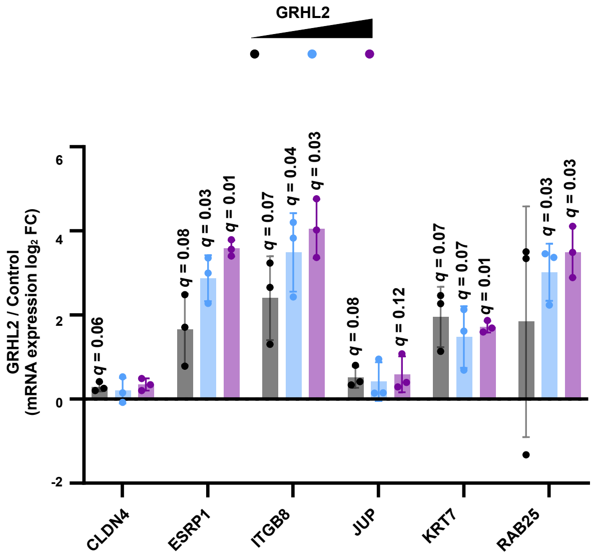

GRHL2
GRHL2 / Control (mRNA expression log₂ FC)
6
4
2
0
-2
q = 0.06
q = 0.08
q = 0.03
q = 0.01
q = 0.07
q = 0.04
q = 0.03
q = 0.08
q = 0.12
q = 0.07
q = 0.07
q = 0.01
q = 0.03
q = 0.03
CLDN4
ESRP1
ITGB8
JUP
KRT7
RAB25

Supplementary Fig.9: Dose-dependent induction of GRHL2 target genes in HepG2 cells

(A) Immunoblotting performed using the Wes system to monitor GRHL2 and HSP90 levels in HepG2 cells transfected with increasing amounts of a GRHL2 expression plasmid or an empty control construct. For GRHL2, reconstructed lane views from the same Wes run using low and high sensitivity settings is shown. Data are representative of those obtained in 2 independents biological replicates. MW, molecular weight.

(B) RT-qPCR data showing the expression of the indicated GRHL2 target genes in HepG2 cells transfected as in panel G (n=3). Log_2 FC in gene expression in cells expressing GRHL2 compared to control cells (transfected with an empty plasmid) are shown in the bar graph, which displays means $\pm$ SD together with individual biological replicates. Two-sided one-sample *t*-test with Benjamini-Hochberg correction was used to determine if the mean of log_2 FC was statistically different from 0. The numerical values of all biological replicates can be found in the S1 data file.
