# Supplementary material for: Derepression of the epithelial transcription factor GRHL2 promotes direct hepatocyte-to-cholangiocyte transdifferentiation
Source: PLoS Biol. 2025 Dec 12;23(12):e3003547. doi: 10.1371/journal.pbio.3003547 (PMC12714216; doi:10.1371/journal.pbio.3003547)
Supplement: S10 Fig — (PDF) [file pbio.3003547.s010.pdf]

Fig.S10

A

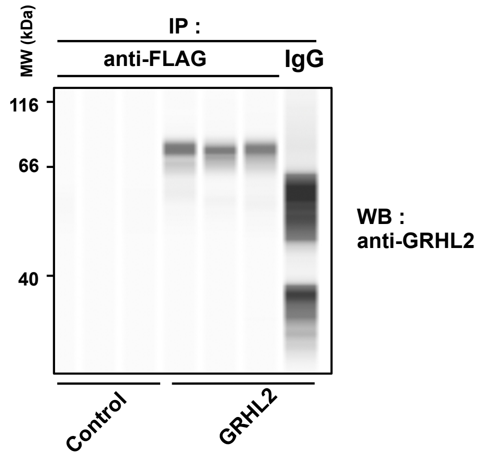
IP :
anti-FLAG
IgG
MW (kDa)
116
66
40
WB :
anti-GRHL2
Control
GRHL2

B

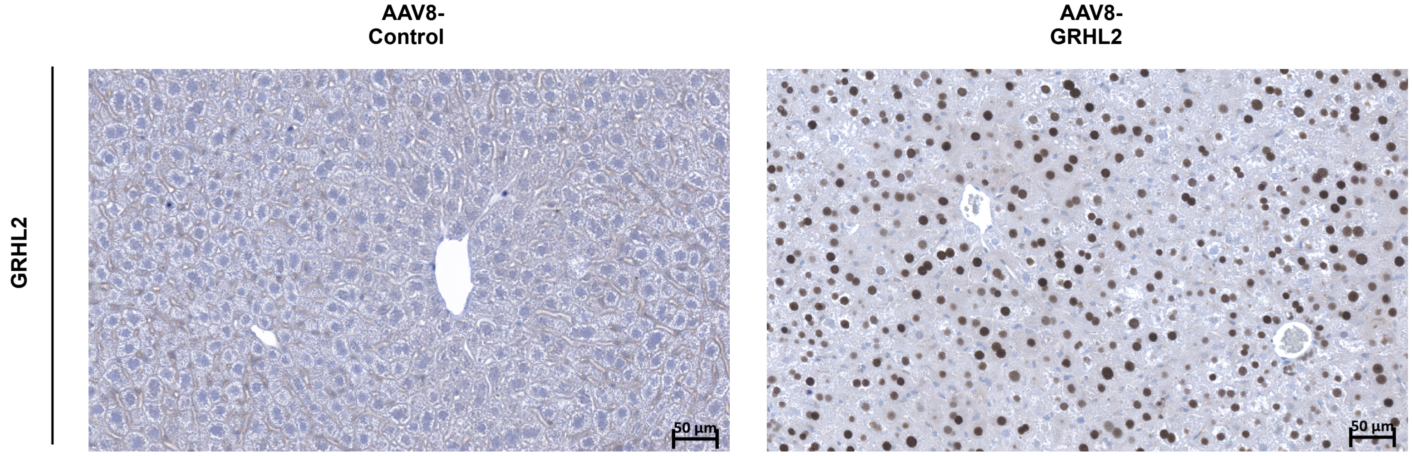
AAV8-
Control
AAV8-
GRHL2
GRHL2
50 µm
50 µm

C

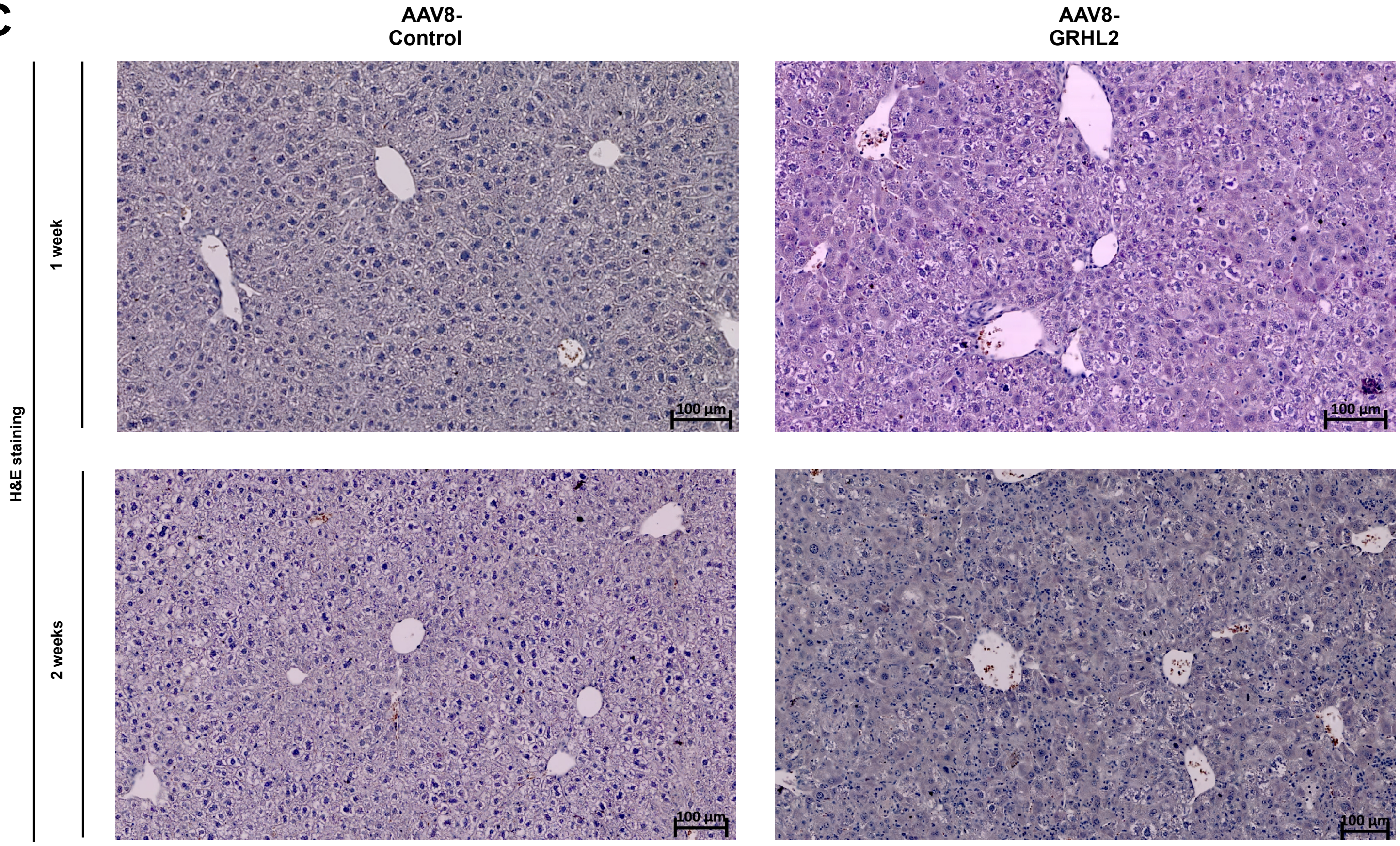
AAV8-
Control
AAV8-
GRHL2
H&E staining
1 week
2 weeks
100 µm
100 µm
100 µm
100 µm

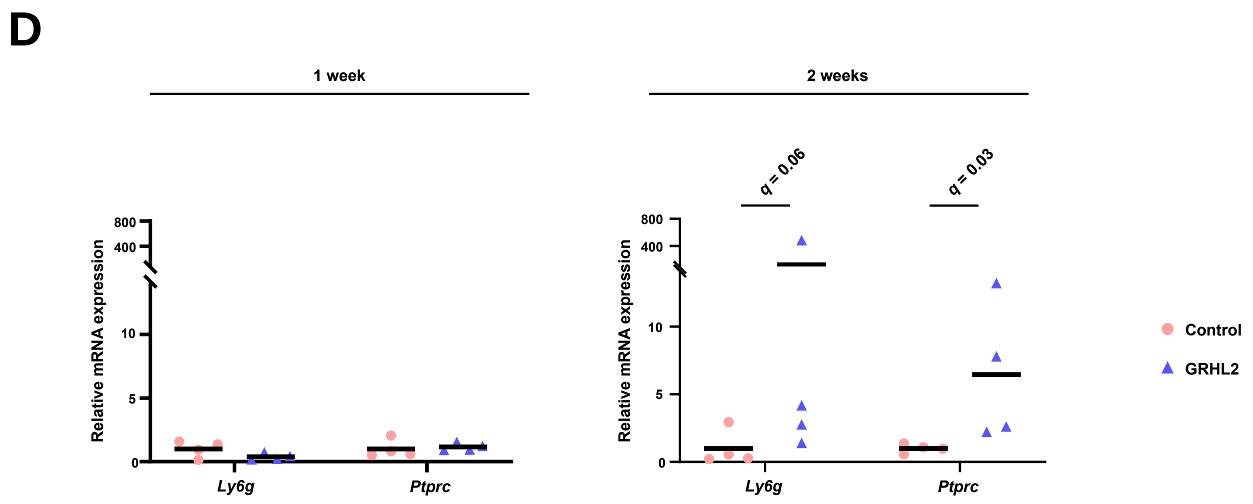
D
1 week
2 weeks
q = 0.06
q = 0.03
800
400
10
5
0
Relative mRNA expression
Ly6g
Ptprc
Control
GRHL2

Supplementary Fig.10: Additional validation of GRHL2 ectopic expression in the mouse liver

(A) Immunoblotting performed using the Wes system to monitor GRHL2 levels in the liver of mice subjected to hydrodynamic injection with the flag-tagged GRHL2-encoding plasmid or an empty construct (control). Nuclear extracts from individual mouse livers (3 mice per experimental group) were subjected to immunoprecipitation with an antibody against flag and immunoblotted using an anti-GRHL2 antibody. As a specificity control, immunoprecipitation with non-immune IgG was also conducted. MW, molecular weight. WB, Wes immunoblotting.

(B) Representative immunostaining of GRHL2 in livers from mice injected with GRHL2 or GFP (control) encoding AAV8 after 1 week.

(C) Representative images obtained using liver slices from mice injected with GRHL2 or GFP (control) encoding AAV8 stained with hematoxylin and eosin after 1 or 2 weeks.

(D) RT-qPCR data showing the relative mRNA expression of *Ly6g* and *Ptprc* (*Cd45*) used as indicators of immune cell infiltration in the liver of mice 1 or 2 weeks after being injected with GRHL2 (n=4 mice) or GFP (control; n=4 mice) encoding AAV8. Data were plotted and analyzed as in Fig.7D. The numerical values of all individual mice can be found in the S1 data file.
