# Supplementary material for: Derepression of the epithelial transcription factor GRHL2 promotes direct hepatocyte-to-cholangiocyte transdifferentiation
Source: PLoS Biol. 2025 Dec 12;23(12):e3003547. doi: 10.1371/journal.pbio.3003547 (PMC12714216; doi:10.1371/journal.pbio.3003547)
Supplement: S11 Fig — (PDF) [file pbio.3003547.s011.pdf]

Fig.S11

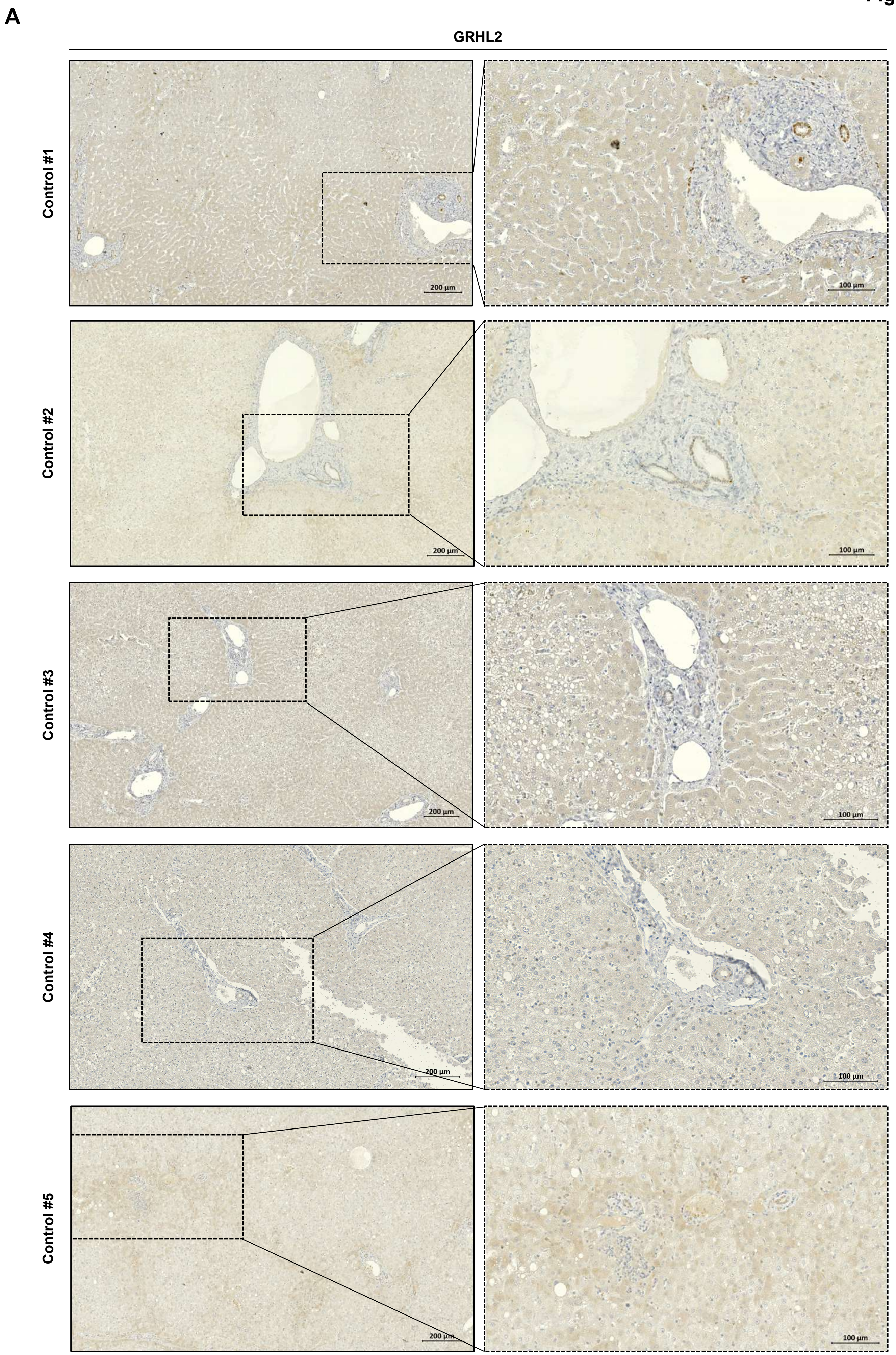
A
GRHL2
Control #1
Control #2
Control #3
Control #4
Control #5
200 µm
100 µm

B

Fig.S11

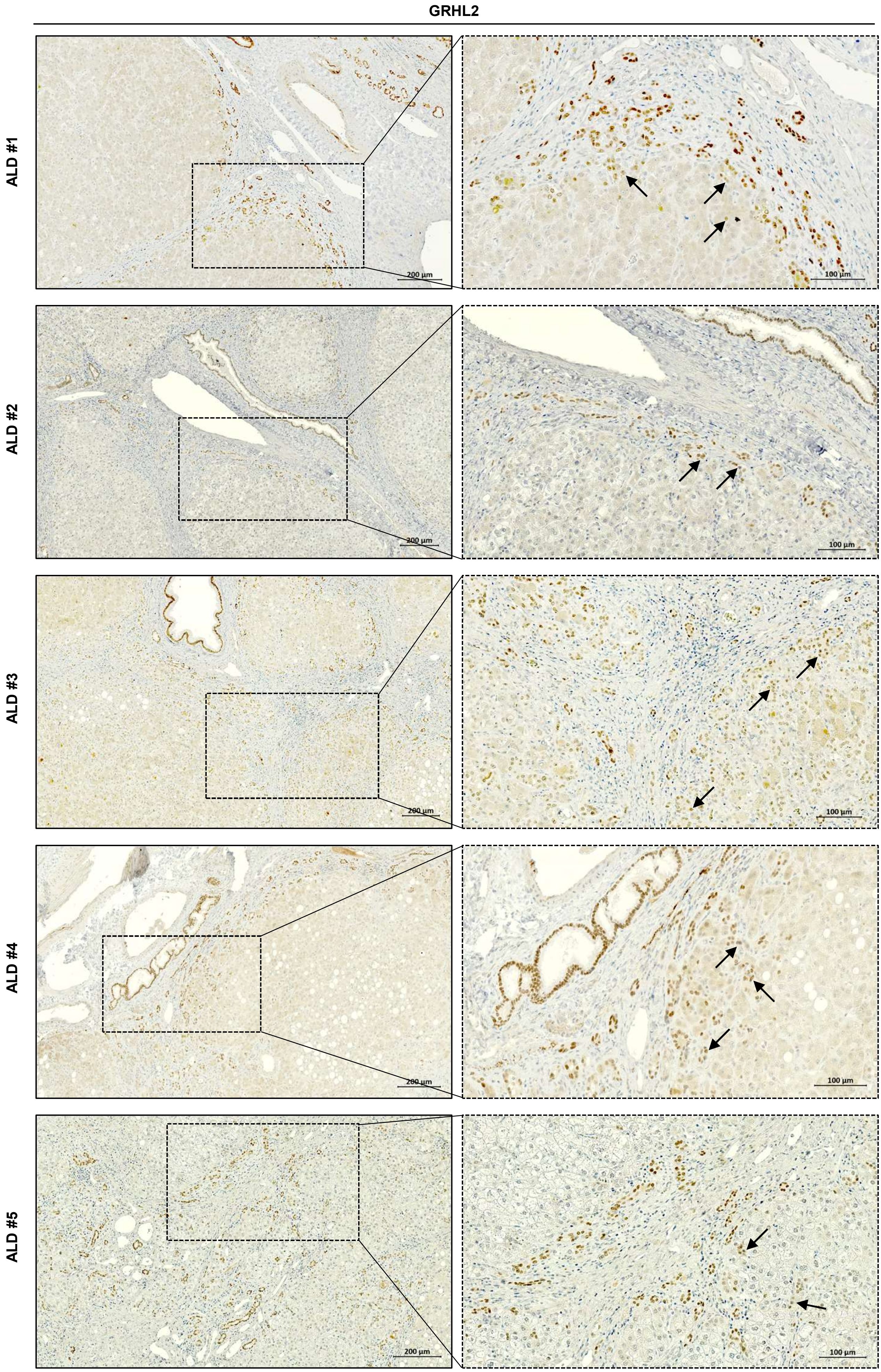

Fig.S11

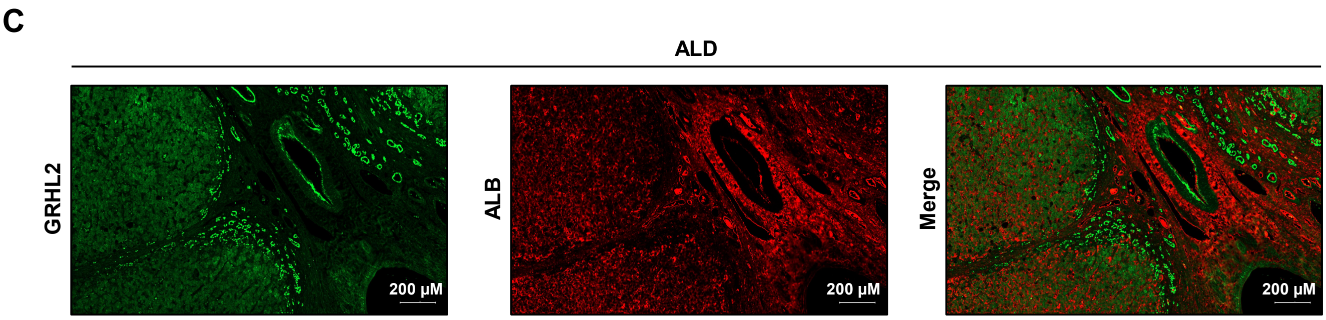
C
ALD
GRHL2
ALB
Merge
200 μM
200 μM
200 μM

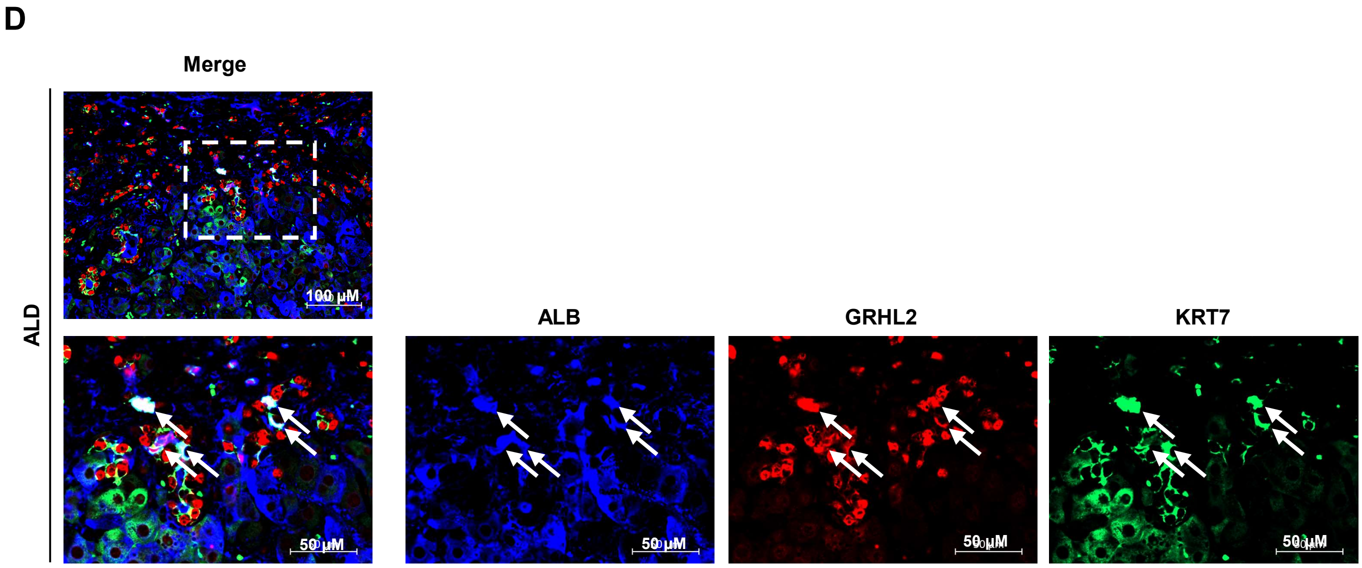
D
Merge
ALD
100 μM
ALB
GRHL2
KRT7
50 μM
50 μM
50 μM
50 μM

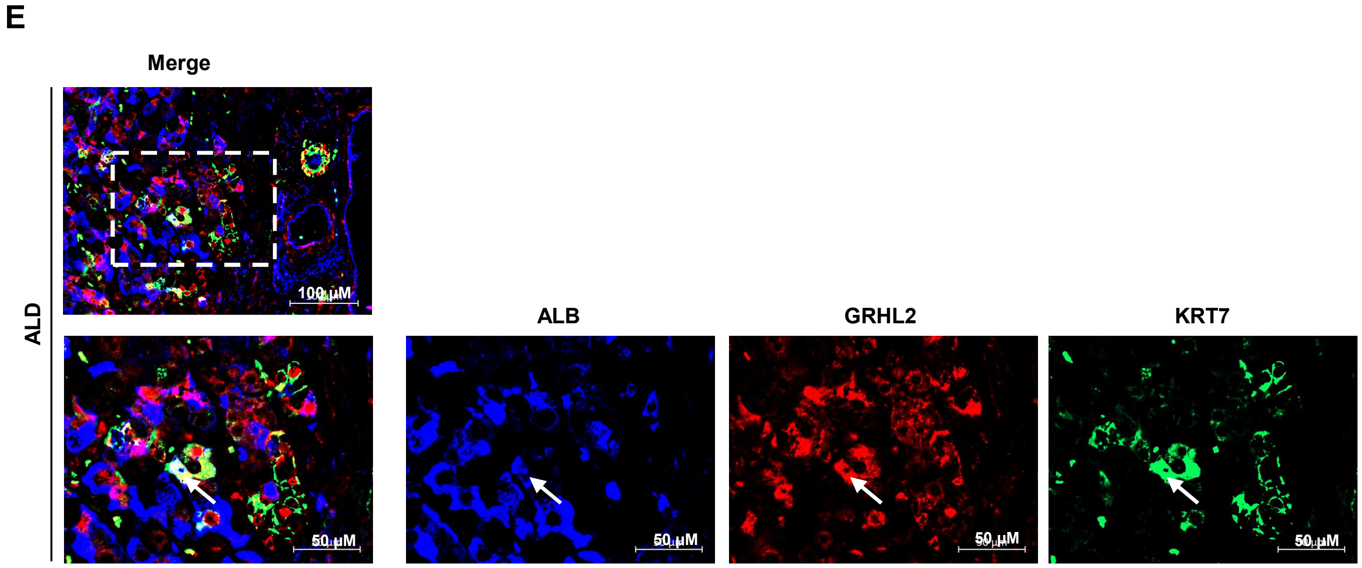
E
Merge
ALD
100 μM
ALB
GRHL2
KRT7
50 μM
50 μM
50 μM
50 μM

Supplementary Fig.11: Additional immunostainings of GRHL2 in human liver failure

(A-B) Immunostaining of GRHL2 in human livers from the control and ALD-related liver failure groups (n=5). Control #1 and ALD #1 are those shown in Fig.8D. Zoomed images of the area delimited by the dotted rectangles is shown on the right. Arrows point to examples of GRHL2-positive hepatocytes.

(C) Co-immunostaining of GRHL2 (in green) and the hepatocyte marker Albumin (ALB; in red) in ALD-related human liver failure performed using a serial section of that shown in Fig.8D.

(D-E) Co-immunostaining of the hepatocyte marker ALB (in blue), GRHL2 (in red) and the cholangiocyte marker KRT7 (in green) in ALD-related human liver failure. Panels D and E show different fields where triple positive cells were observed (indicated by arrows). Zoomed images of the area delimited by the dotted rectangle in the top image are shown at the bottom.
