# Supplementary material for: Derepression of the epithelial transcription factor GRHL2 promotes direct hepatocyte-to-cholangiocyte transdifferentiation
Source: PLoS Biol. 2025 Dec 12;23(12):e3003547. doi: 10.1371/journal.pbio.3003547 (PMC12714216; doi:10.1371/journal.pbio.3003547)
Supplement: S12 Fig — (PDF) [file pbio.3003547.s012.pdf]

Fig.S12

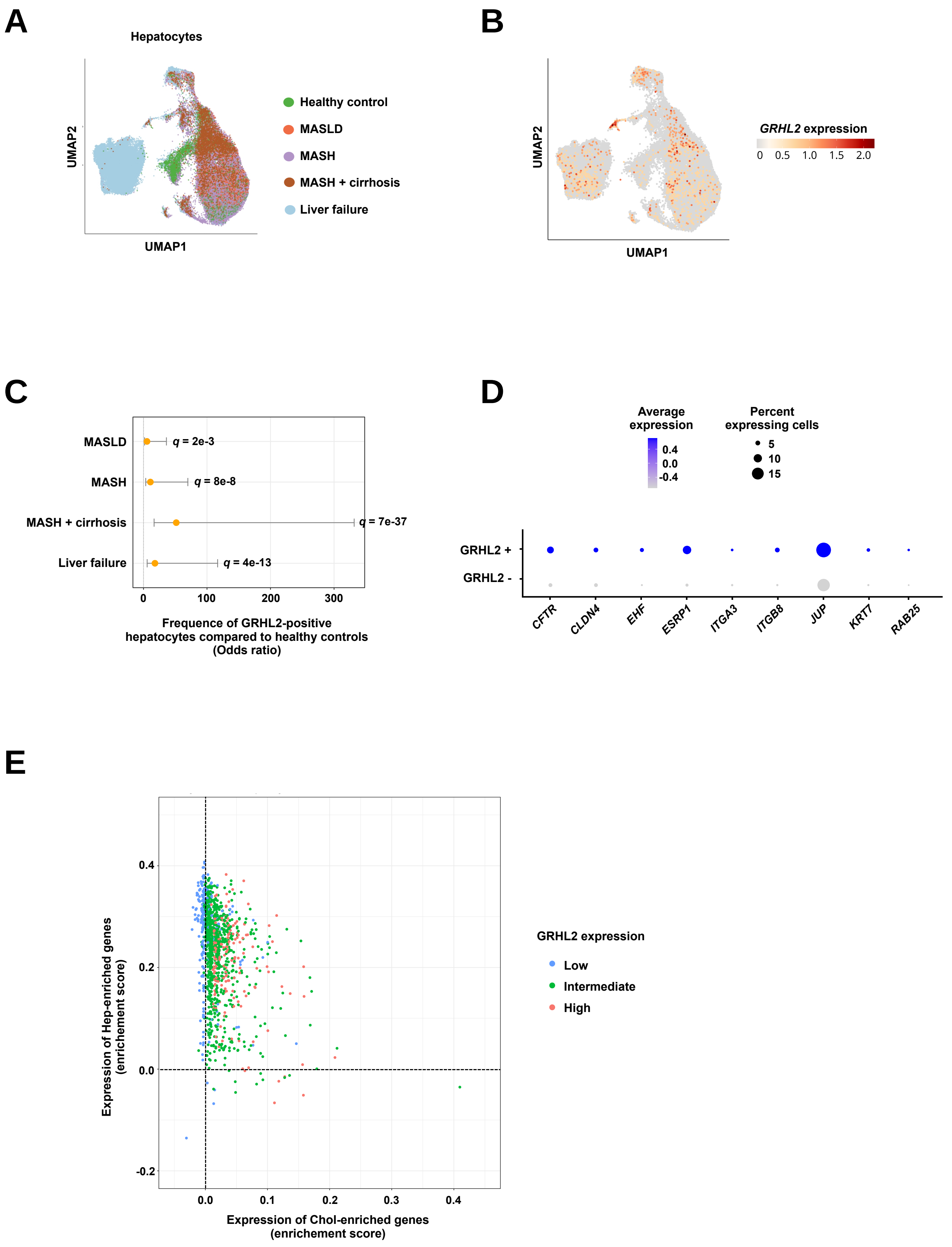

A
Hepatocytes
UMAP2
UMAP1
Healthy control
MASLD
MASH
MASH + cirrhosis
Liver failure
B
UMAP2
UMAP1
GRHL2 expression
0 0.5 1.0 1.5 2.0
C
MASLD
MASH
MASH + cirrhosis
Liver failure
q = 2e-3
q = 8e-8
q = 7e-37
q = 4e-13
0 100 200 300
Frequence of GRHL2-positive hepatocytes compared to healthy controls (Odds ratio)
D
Average expression
0.4
0.0
-0.4
Percent expressing cells
5
10
15
GRHL2 +
GRHL2 -
CFTR
CLDN4
EHF
ESRP1
ITGA3
ITGB8
JUP
KRT7
RAB25
E
0.4
0.2
0.0
-0.2
0.0 0.1 0.2 0.3 0.4
Expression of Hep-enriched genes (enrichement score)
Expression of Chol-enriched genes (enrichement score)
GRHL2 expression
Low
Intermediate
High

Supplementary Fig.12: Characterization of GRHL2-expressing hepatocytes in human MASLD using snRNA-seq data

(A-B) Data from (Gribben et al. 2024) were used to monitor *GRHL2* expression in hepatocytes obtained from human livers of MASLD patients. Panel A shows the different stages of the disease hepatocyte originate from while panel B shows *GRHL2* expression in single nuclei.

(C) The fraction of *GRHL2*-expressing hepatocytes at each disease stage was retrieved and odds ratios were computed using hepatocytes from healthy controls as the reference. The forest plot shows odds ratio values (orange dots) together with 95 % confidence intervals. Statistical significance was assessed using two-sided Fisher exact tests with with Benjamini-Hochberg correction.

(D) Bubble plots showing expression of the indicated genes in hepatocytes negative (*GHRL2* -) or positive (*GRHL2* +) for *GRHL2* expression (i.e. any hepatocytes from panel A where *GRHL2* expression was detected).

(E) *GRHL2*-positive hepatocytes were assessed for expression of the Chol-enriched or Hep-enriched gene sets using the AddModuleScore function of Seurat. Individual cells are colored according to *GRHL2* expression levels (i.e. tertiles defined as low, intermediate and high expression).
