# Supplementary material for: Derepression of the epithelial transcription factor GRHL2 promotes direct hepatocyte-to-cholangiocyte transdifferentiation
Source: PLoS Biol. 2025 Dec 12;23(12):e3003547. doi: 10.1371/journal.pbio.3003547 (PMC12714216; doi:10.1371/journal.pbio.3003547)
Supplement: S13 Fig — (PDF) [file pbio.3003547.s013.pdf]

A **Fig.S13**

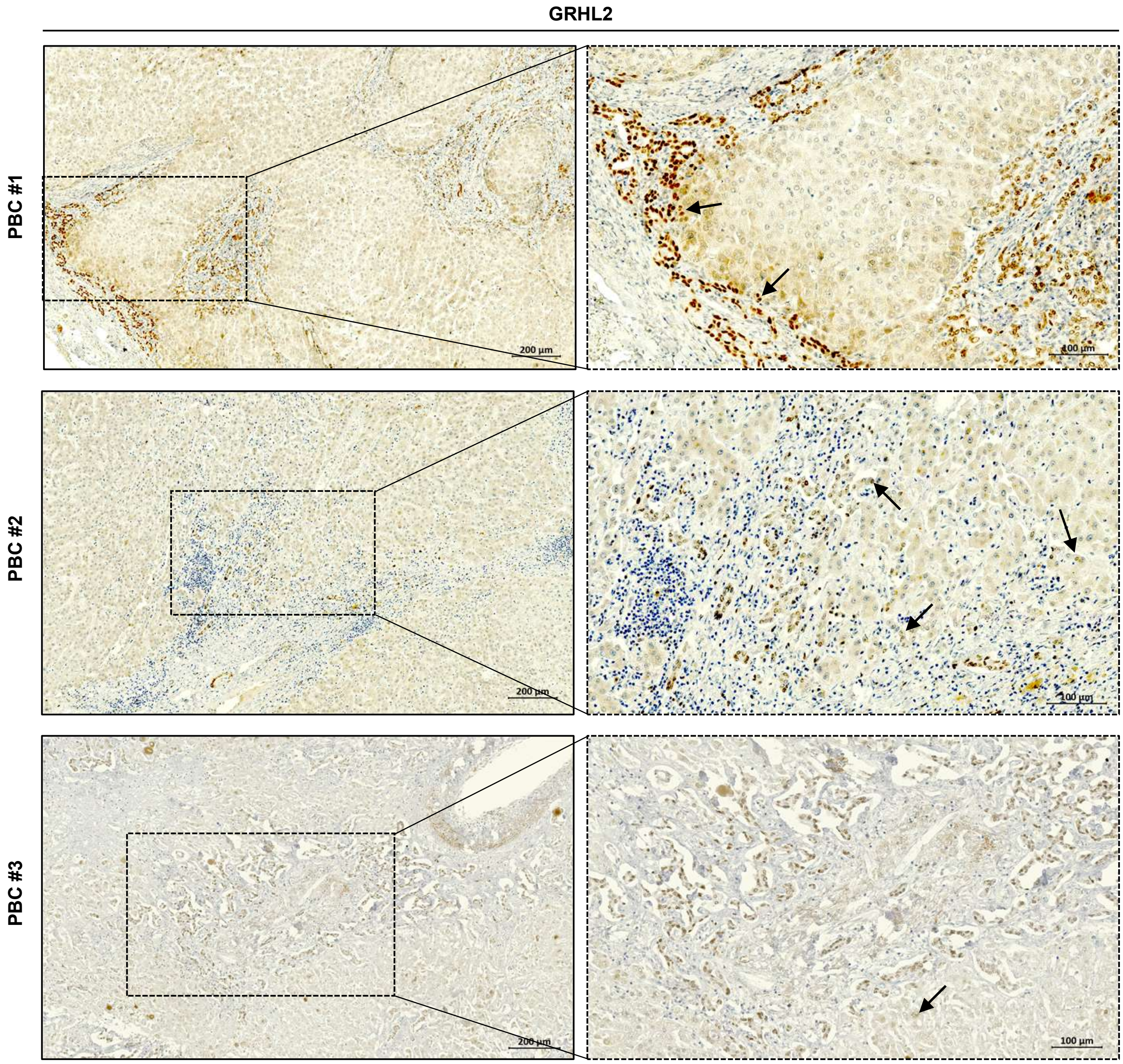

Fig.S13

B

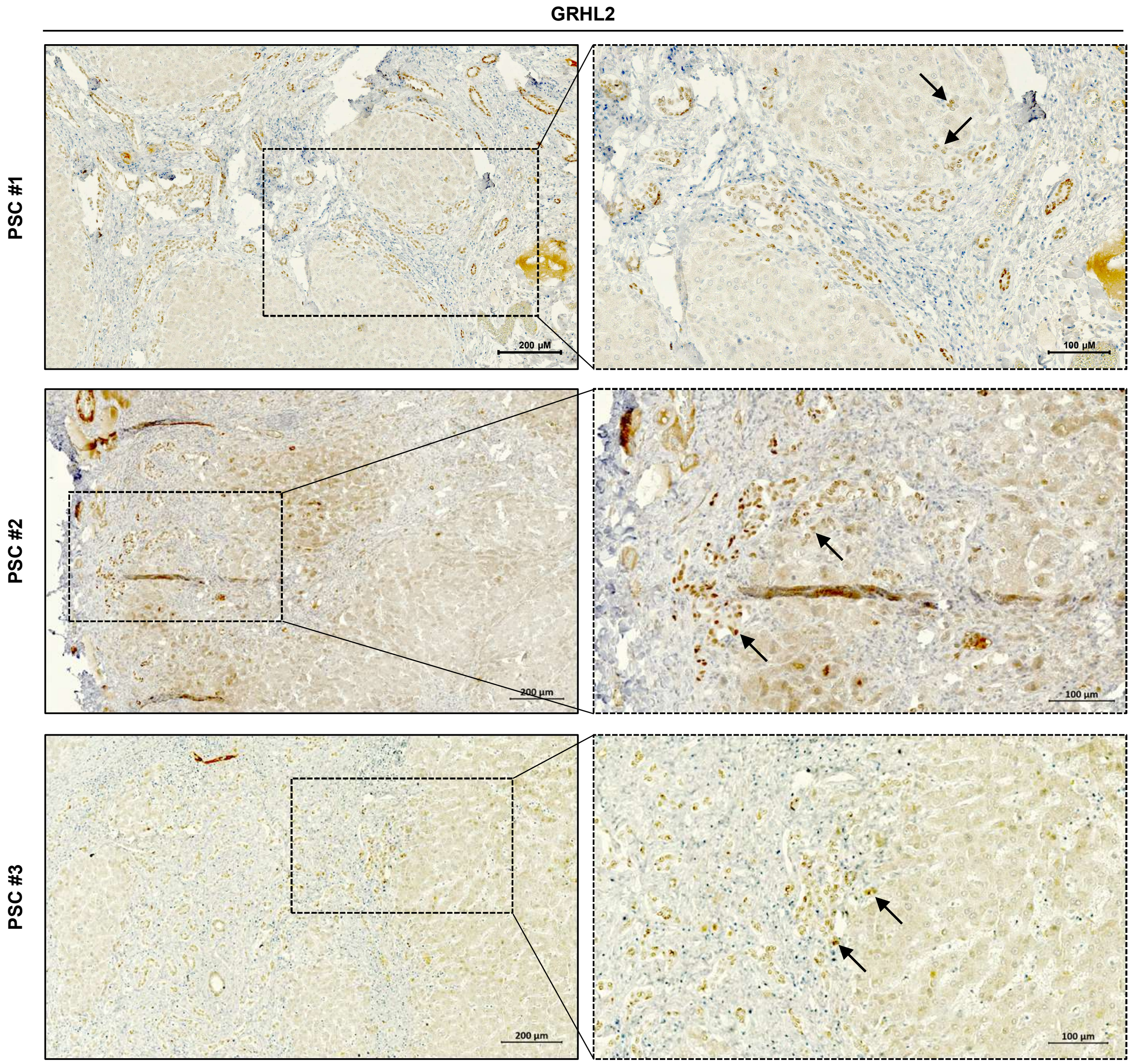

Supplementary Fig.13: Additional immunostainings of GRHL2 in human PBC and PSC samples

(A-B) Immunostaining of GRHL2 in human livers from donors with PBC- or PSC-related liver failure (n=3 each). PBC #1 and PSC #1 are those shown in Fig.8F. Zoomed images of the area delimited by the dotted rectangles is shown on the right. Arrows point to examples of GRHL2-positive hepatocytes.

References

Bou Saleh M, Louvet A, Ntandja-Wandji LC, Boleslawski E, Gnemmi V, Lassailly G, Truant S, Maggiotto F, Ningarhari M, Artru F et al. 2021. Loss of hepatocyte identity following aberrant YAP activation: a key mechanism in alcoholic hepatitis. *J Hepatol* **75**: 912-923.

Bravo Gonzalez-Blas C, Matetovici I, Hillen H, Taskiran, II, Vandepoel R, Christiaens V, Sansores-Garcia L, Verboven E, Hulselmans G, Poovathingal S et al. 2024. Single-cell spatial multi-omics and deep learning dissect enhancer-driven gene regulatory networks in liver zonation. *Nat Cell Biol* **26**: 153-167.

Breschi A, Muñoz-Aguirre M, Wucher V, Davis CA, Garrido-Martín D, Djebali S, Gillis J, Pervouchine DD, Vlasova A, Dobin A et al. 2020. A limited set of transcriptional programs define major cell types. *Genome Research* **30**: 1047-1059.

Dubois-Chevalier J, Gheeraert C, Berthier A, Boulet C, Dubois V, Guille L, Fourcot M, Marot G, Gauthier K, Dubuquoy L et al. 2023. An extended transcription factor regulatory network controls hepatocyte identity. *EMBO Rep* doi:10.15252/embr.202357020: e57020.

Gribben C, Galanakis V, Calderwood A, Williams EC, Chazarra-Gil R, Larraz M, Frau C, Puengel T, Guillot A, Rouhani FJ et al. 2024. Acquisition of epithelial plasticity in human chronic liver disease. *Nature* doi:10.1038/s41586-024-07465-2.

Grindheim JM, Nicetto D, Donahue G, Zaret KS. 2019. Polycomb Repressive Complex 2 Proteins EZH1 and EZH2 Regulate Timing of Postnatal Hepatocyte Maturation and Fibrosis by Repressing Gene Expression at Promoter Regions in Euchromatin in Mice. *Gastroenterology* doi:10.1053/j.gastro.2019.01.041.

Merrell AJ, Peng T, Li J, Sun K, Li B, Katsuda T, Grompe M, Tan K, Stanger BZ. 2021. Dynamic transcriptional and epigenetic changes drive cellular plasticity in the liver. *Hepatology* doi:10.1002/hep.31704.

Schaub JR, Huppert KA, Kurial SNT, Hsu BY, Cast AE, Donnelly B, Karns RA, Chen F, Rezvani M, Luu HY et al. 2018. De novo formation of the biliary system by TGFβ-mediated hepatocyte transdifferentiation. *Nature* doi:10.1038/s41586-018-0075-5.

Zhang C, Macchi F, Magnani E, Sadler KC. 2021. Chromatin states shaped by an epigenetic code confer regenerative potential to the mouse liver. *Nat Commun* **12**: 4110.

Zummo FP, Berthier A, Gheeraert C, Vinod M, Bobowski-Gerard M, Molendi-Coste O, Pineau L, Jung M, Guille L, Dubois-Chevalier J et al. 2023. A time- and space-resolved nuclear receptor atlas in mouse liver. *J Mol Endocrinol* **71**.
