# Supplementary material for: Derepression of the epithelial transcription factor GRHL2 promotes direct hepatocyte-to-cholangiocyte transdifferentiation
Source: PLoS Biol. 2025 Dec 12;23(12):e3003547. doi: 10.1371/journal.pbio.3003547 (PMC12714216; doi:10.1371/journal.pbio.3003547)

Fig.4E – Immunoblotting on WES system on HepG2 treated or not with Ezhi

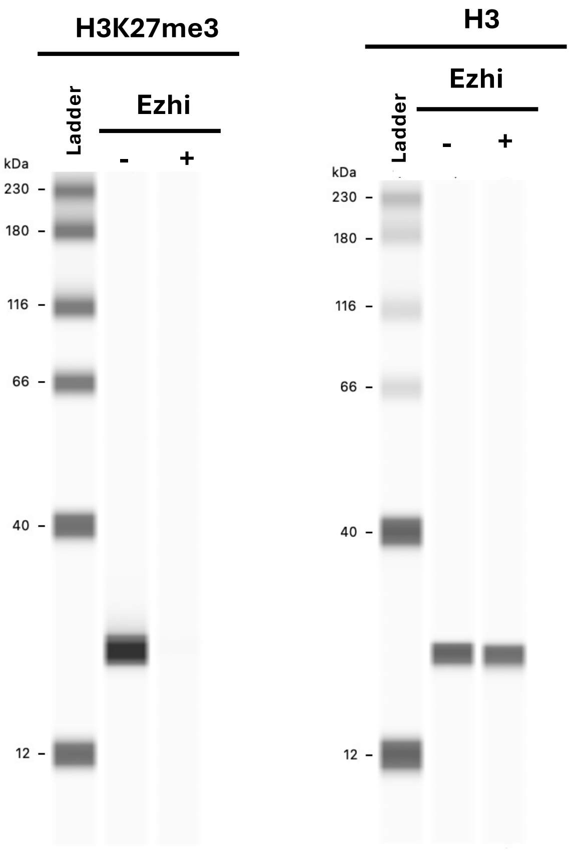

Fig.4D – Immunoblotting on WES system on BMEL cells transfected with GRHL2 plasmid or empty control

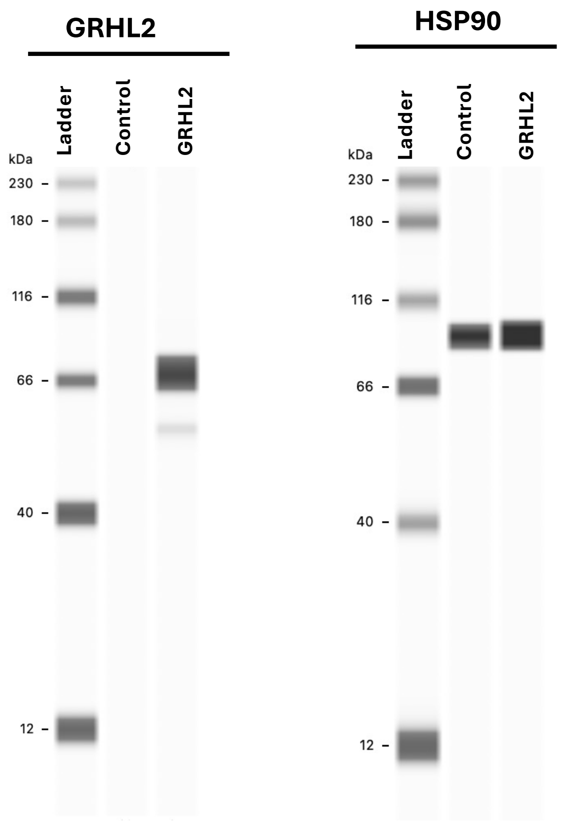

Fig.6A – Immunoblotting on WES system on HepG2 cells transfected with GRHL2 plasmid or empty control

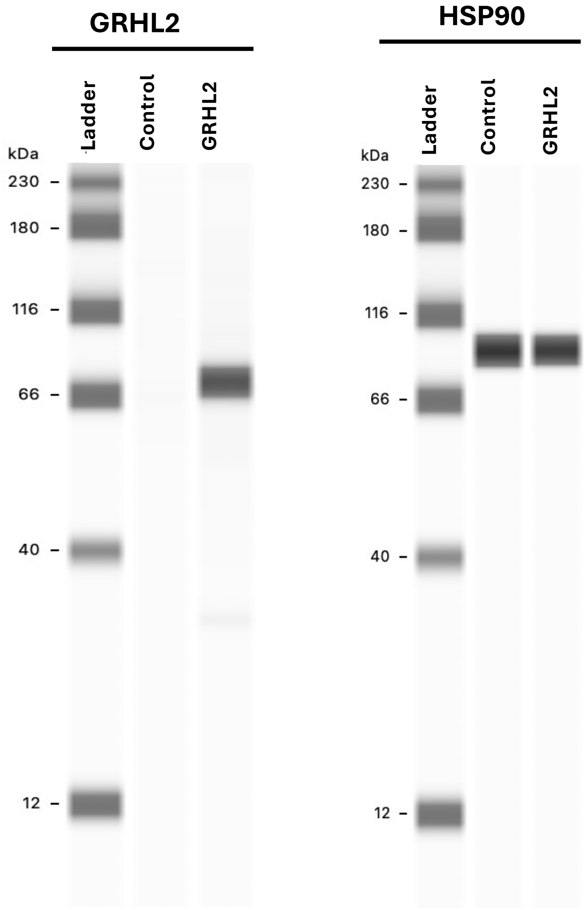

Fig.6H – Immunoblotting on WES system on HepaRG cells transfected with GRHL2 plasmid or empty control

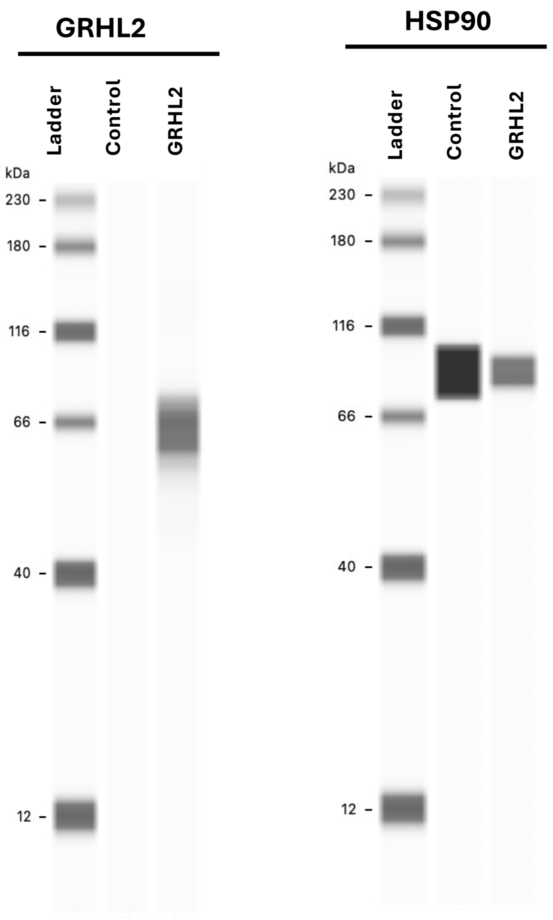

Fig.7A – Immunoblotting on WES system on the liver of mice subjecting to hydrodynamic injection

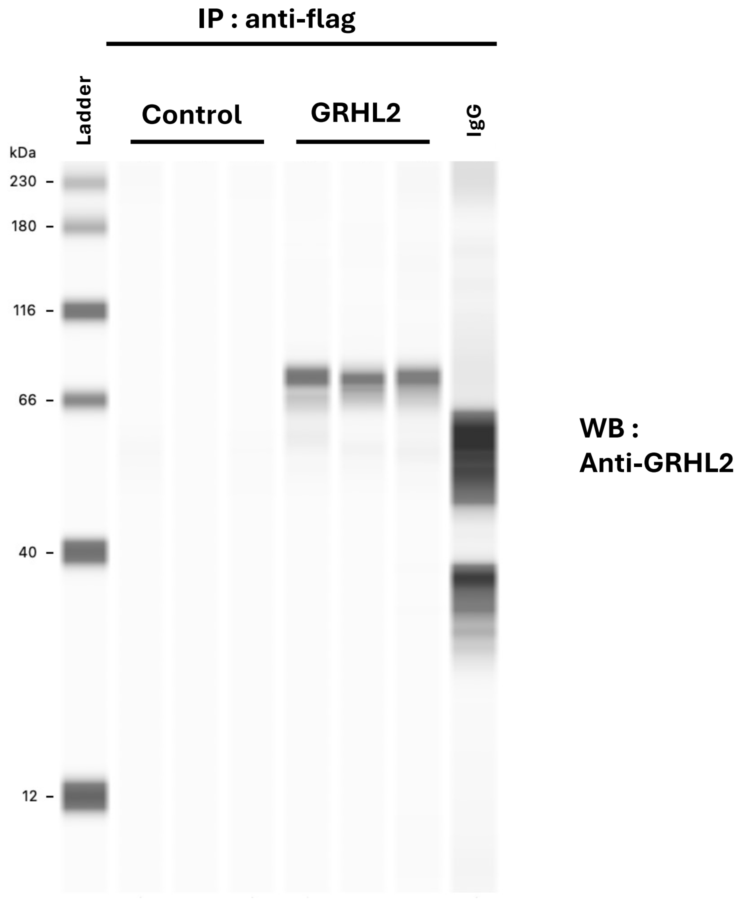

Fig.7B – Immunoblotting on WES system on the liver of mice injecting with GRHL2 or GFP encoding AAV8

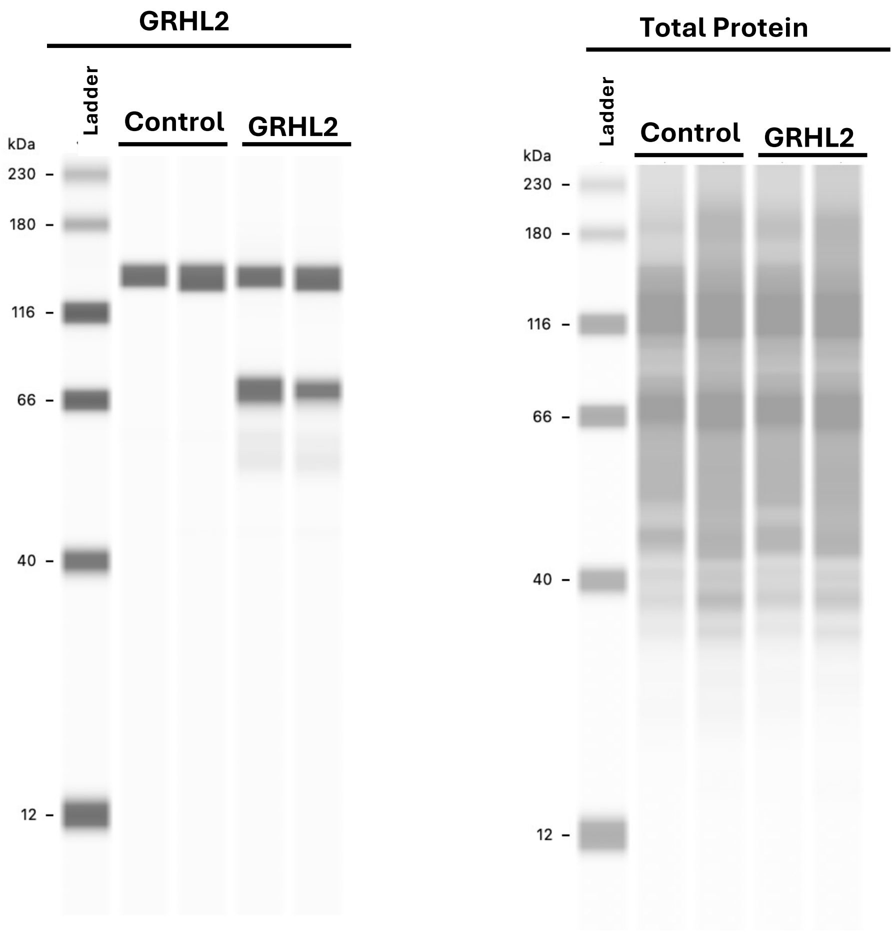

Fig.8B – Immunoblotting on WES system of human samples from control and ALD-related liver failure

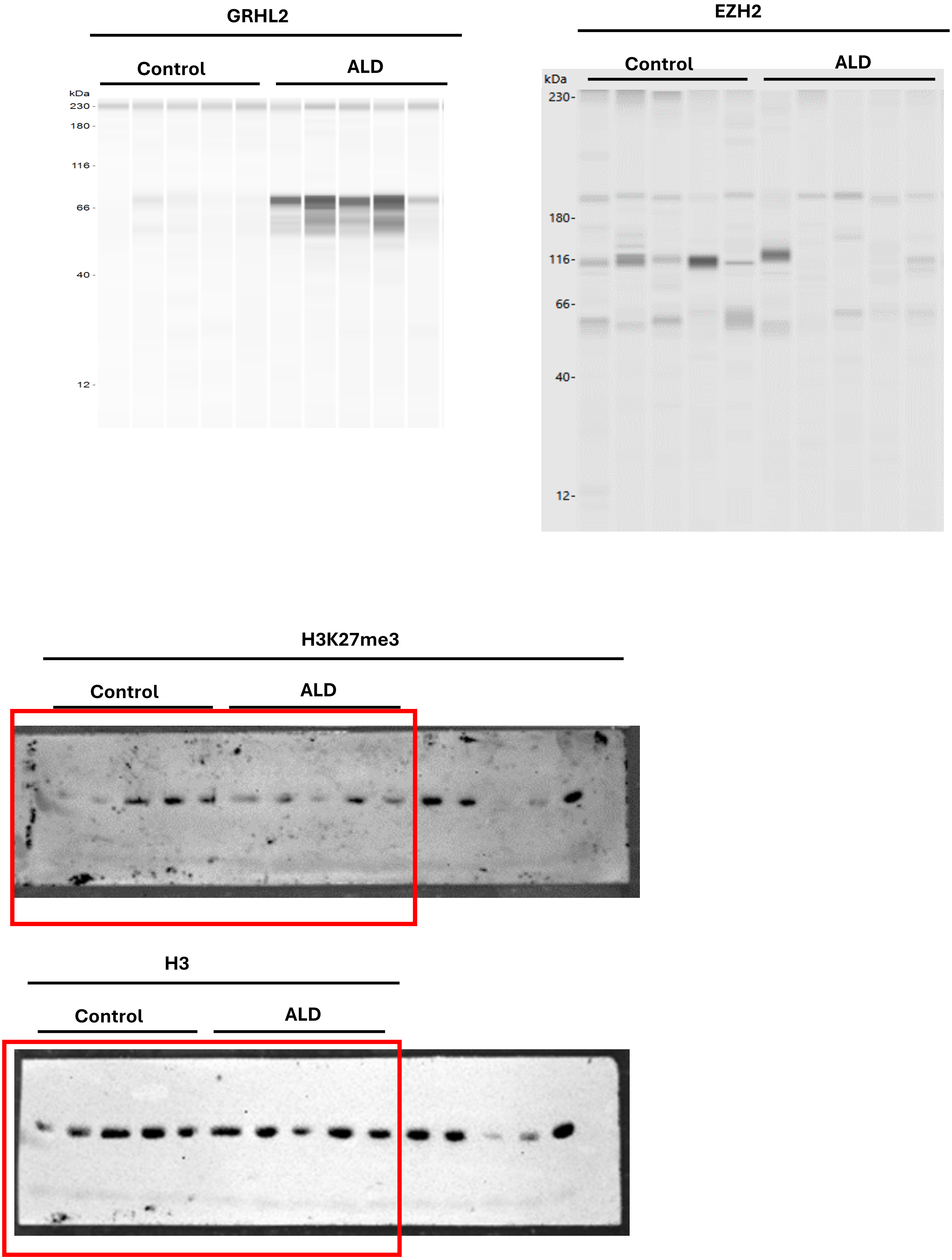

Fig.S7A – Immunoblotting on WES system on BMEL cells transfected with GRHL2 plasmid or empty control

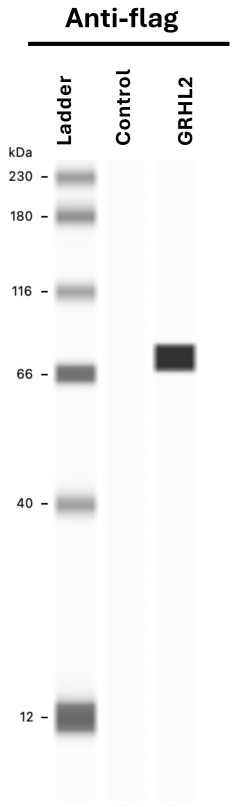

Fig.S7C – Immunoblotting on WES system on HepG2 cells transfected with GRHL2 plasmid or empty control

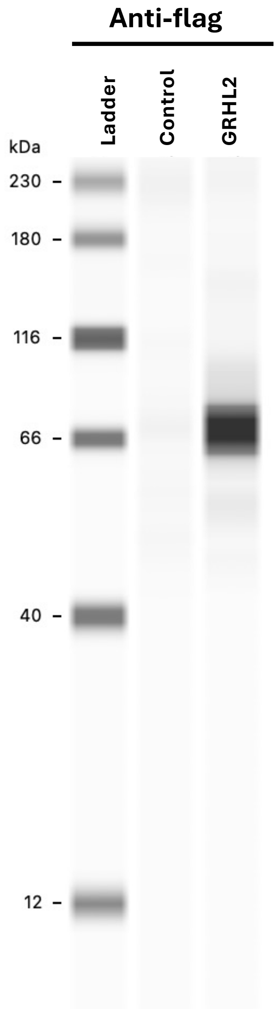

Fig.S7G – Immunoblotting on WES system on HepG2 cells transfected with GRHL2 plasmid or empty control

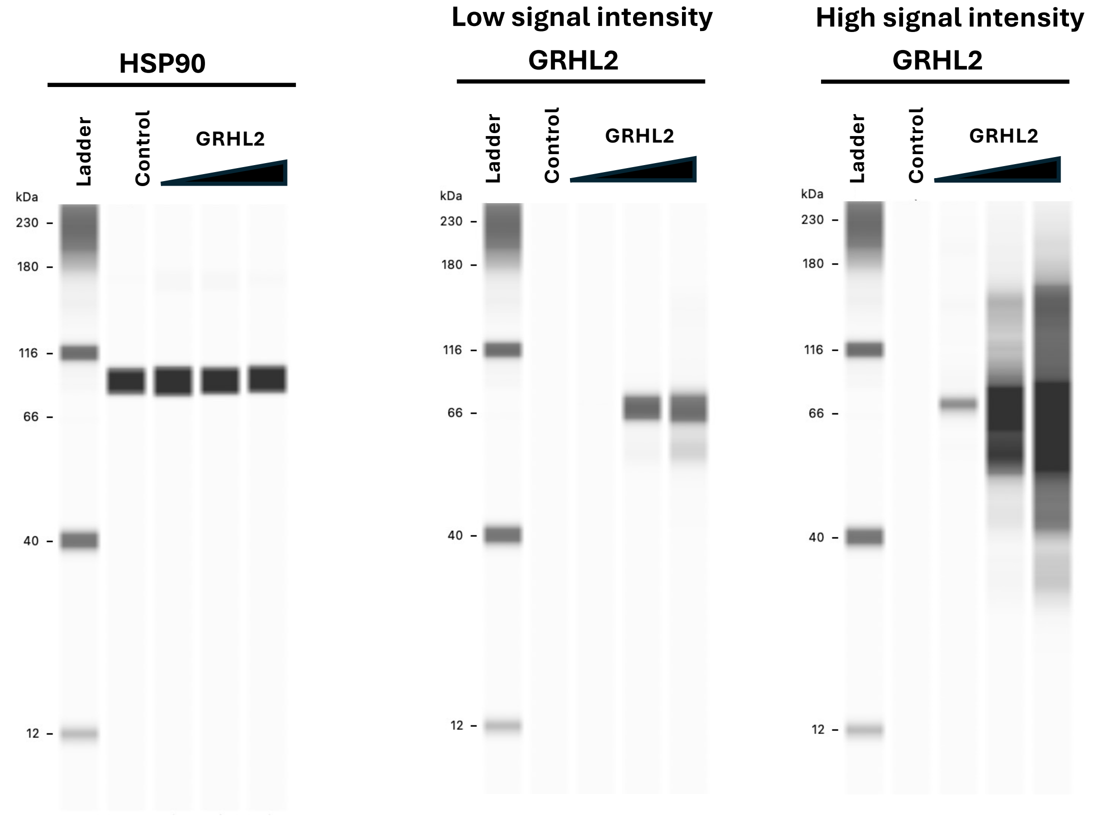

Supplement: S1 Raw Images — (PDF) [file pbio.3003547.s027.pdf]
