# Supplementary material for: Derepression of the epithelial transcription factor GRHL2 promotes direct hepatocyte-to-cholangiocyte transdifferentiation
Source: PLoS Biol. 2025 Dec 12;23(12):e3003547. doi: 10.1371/journal.pbio.3003547 (PMC12714216; doi:10.1371/journal.pbio.3003547)
Supplement: Attachment — Submitted filename: 2025_08_29_Rebuttal.docx [file pbio.3003547.s030.docx]

We would like to thank the reviewers and editors for their constructive evaluation of our manuscript. As detailed in our point-by-point response below, we have performed additional experiments and analyses and refined the discussion to strengthen the manuscript in line with the editors’ and reviewers’ suggestions.

**Reviewer #1**, Holger Willenbring (note, reviewer 1 has signed this review): The manuscript by Vasseur et al investigates epigenetic mechanism of hepatocyte-to-cholangiocyte transdifferentiation, which has been identified as a response to liver injury in both mice and humans. The authors make good use of published datasets to arrive at the compelling conclusion that hepatocyte-to-cholangiocyte transdifferentiation is, at its core, the introduction of a generic epithelial transcriptional program at the expense of the unique transcriptome and specification characteristic for hepatocytes. The authors show that this process does not involve a progenitor-like intermediate as suggested by others. They identify an epigenetic mechanism regulating this process, that is, loss of EZH1/2 expression freeing epithelial transcription factor genes such as GRHL2 from repression by H3K27me3. Their conclusions rest on multiple lines of evidence, including functional testing in several liver cell
lines. The data are clearly presented and described, and rounded out by several tables. There are only a couple of places where the descriptions seem incomplete or off.

1.	In the results on page 13 it is not totally clear what genes constitute the "cholangiocyte-like transcriptional program", that is, what genes are induced by GRHL2 beyond established downstream targets like RAB25 and CLDN4 and the four others genes that are mentioned. This deficiency could be addressed by highlighting genes in Fig. S7B, D and expanding Supplementary Table 5. Along these lines, the authors mention SOX4 on page 18 but it does not seem to feature in any of the figures.

In the revised manuscript, we more clearly define that acquisition of a cholangiocyte-like transcriptional program critically involves induction of genes defining archetypal epithelial cells (pages 7-9). This was captured through induction of the Epith-Signature during HCT (Fig.2C-D). Additional analyses indicate that the Epith-Signature comprises more than 60% of the genes with enriched expression in cholangiocytes (when compared to other liver cell types) including commonly used cholangiocyte markers (Fig.2E described page 8; gene lists for the Epith-Signature and Chol-enriched gene sets now included in Supplementary Table 2). We concluded from these analyses that induction of a cholangiocyte-like program critically involves activation of a *bone fide* epithelial gene program (page 9).

In this context, in addition to providing the full list of GHRL2-induced genes in BMEL and HepG2 cells issued from our RNA-seq analyses (new Supplementary Tables 6 and 8), we now also point to individual GRHL2 target genes from the Epith-Signature (Supplementary Fig.7B, D and E). Among those genes, we now additionally highlight *ESRP1* and *KRT7* are direct GRHL2 targets (Fig.6 and 7; pages 13-14) extending the set of key epithelial/cholangiocyte identity genes whose expression is stimulated by GRHL2.

With regards to SOX4, we now indicate page 18 that it belongs to the list of transcription factors induced during HCT provided in Supplementary Table 3.

2.	In the discussion on page 18 the authors refer to a paper by Yang et al from 2023 as being "at apparent odds with our findings". However, that paper shows that EZH2 inhibition or knockout results in more EPCAM-positive cells (that is, cholangiocytes) in E12.5 liver slices (Fig. 5B-D) or in mice at E17.5 and P0 (Fig. 6A-D), which is associated with reduced H3K27me3 levels and therefore seems consistent with the results by Vasseur et al.

We thank the reviewer for drawing our attention to this confusing statement, which was corrected as follows: “A role for H3K27me3 in controlling hepatoblast-to-cholangiocyte differentiation has been reported in a study showing that EZH2 inhibition promotes cholangiocyte differentiation [93]. This is in line with our findings that loss of EZH2-mediated repression is involved in HCT, even though HCT does not *per se* phenocopy the developmental process.” (page 19).

**Reviewer #2**, Pinglong Xu (note, reviewer 2 has signed this review): The manuscript titled "Direct hepatocyte-to-cholangiocyte transdifferentiation driven by derepression of the epithelial transcription factor GRHL2" by Eeckhoute et al. presents interesting findings that adult hepatocytes can convert directly into cholangiocytes (HCT) by relieving polycomb-mediated epigenetic repression of epithelial transcriptional regulator GRHL2. The authors propose that HCT bypasses a hepatoblast-like state, instead triggering a monopolarized epithelial gene program upon GRHL2 induction, thereby suggesting that derepressing an epithelial transcription factor is sufficient for lineage reprogramming and providing implications in human liver diseases.
Understanding HCT is crucial for liver regeneration and for understanding chronic liver diseases. These findings highlight the importance of the repression of the epithelial transcription program during the HCT transition. The manuscript presents a convincing data analysis supporting the conclusions drawn. However, a few key issues remain unaddressed:

1.      The manuscript provides strong gain-of-function evidence that overexpression of GRHL2 in hepatocytes can induce epithelial gene expression and promote HCT both in vitro and in vivo. However, the proposed mechanism remains incomplete due to the lack of rigorous loss-of-function data. Will depleting or inhibiting GRHL2 interrupt the HCT process, such as during the in vitro HCT system and with a hepatocyte-specific knockout of GRHL2 by AAV or other systems?

We thank the reviewer for this suggestion. Despite its recognized interest, we noted that the editors did not require us to provide additional loss-of-function data. In compliance with the editorial recommendations, we did not engage in this line of research but rather strengthened the manuscript by refining the description of the mechanisms underlying GHRL2-mediated HCT. For instance, we provide in the revised manuscript greater details regarding GRHL2 target genes and show their induction is achieved with moderate GRHL2 ectopic expression (Fig.6-7 and Supplementary Fig.9). Moreover, we have further linked GRHL2-mediated gene induction to acquisition of a bile-duct phenotype and repression of the hepatocyte program using additional experiments in human organoids and in vivo in the mouse liver (Fig.5-6-7). Finally, we more directly show EZH1-2/H3K27me3-mediated repression of the *GRHL2* gene (Fig.5). A detailed description of all newly acquired data, which strengthen the role of GRHL2 in the induction of HCT, can be found hereafter and in response to the other reviewers’ comments.

2.      How precisely PRC2 and H3K27me3 target GRHL2 and other "epithelial" genes in hepatocytes is unclear. Is there direct evidence that modulating EZH2 or PRC2 activity can promptly derepress GRHL2?

We now show more clearly that EZH2 binds to the *Grhl2* gene promoter in the mouse liver. This is accompanied by the presence of H3K27me3 at this promoter in hepatocytes while, as expected, this repressive mark is not observed at the *Grhl2* gene in cholangiocytes. Moreover, *Ezh1/2* deletion in hepatocytes *in vivo* leads to both a reduced H3K27me3 signal at the *Grhl2* promoter and *Grhl2* induction. These new data are shown in Fig.5C and described on page 12.

More generally, a direct role for EZH2 and H3K27me3 in the repression of TF encoding genes from C3 (TFs repressed in hepatocytes and up-regulated upon HCT) is now also shown in Fig.4A-C which includes a new panel B showing decreased H3K27me3 at the promoter of these genes in the liver of mice with hepatocyte-specific deletion of *Ezh1/2*. As a consequence, we found that the Epith-Signature genes are induced in the liver of mice with hepatocyte *Ezh1/2* deletion (new panel shown in Fig.4D; described page 11).

3.      Beyond changes in transcript profiles, do these cells acquire bile duct-like structures or secretory functions to confirm full cholangiocyte functionality?

To assess the effect of GRHL2 on the acquisition of a cholangiocyte-like cell phenotype, we grew BMEL cells overexpressing GRHL2 in 3D in Matrigel. We observed that GRHL2 promotes the acquisition of a cyst-like organization by BMEL cells, reminiscent of how cholangiocytes and epithelial cells behave in three-dimensional cell culture (Fig.5G-H) (PMID22696678 and PMID33961769). In human livers from patients with alcoholic hepatitis, we found GRHL2-positive hepatocytes at the interface with scar tissues (in line with the spatial transcriptomic data recently reported in PMID40281306) where these cells are found to adopt cyst-like structures (Fig.8 and Supplementary Fig.9). Additionally, GRHL2 and TFs from the C3 clusters are also induced in hepatocytes converting into mature cholangiocytes in a mouse model of ALGS (Fig.3B). These cholangiocytes derived from hepatocytes were shown to form bile ducts that are effective in draining bile and persist after the cholestatic liver injury is reversed, consistent with transdifferentiation (PMID29720662). Novel experiments where we overexpressed GRHL2 in hepatocytes *in vivo* led to a dramatic alteration in the hepatic parenchyma structure underlain by loss of the archetypal hepatocyte cell shape and dampened expression of hepatocyte identity genes (Fig.7 and Supplementary Fig.8). Together with the fact that epithelial genes directly induced by GRHL2 define a main event in the acquisition of a cholangiocyte-like transcriptional program (see the response to Reviewer 1’s point 1 and Reviewer 3’s point 3), these observations point to GRHL2 driven HCT as a genuine transdifferentation event.

4.      Fig. 6B, the correlation between GRHL2 protein levels and H3K27me3/EZH2 is weak in individual samples.

The use of human samples in research is subjected to several limitations, often resulting in greater data heterogeneity compared to controlled animal or *in vitro* model experiments. Here, for instance, control samples are non-tumoral liver zones distant from resected metastatic nodules. While this corresponds to the commonly used strategy to obtain human samples reflecting as close as possible the healthy liver. Heterogeneity within the liver sample, in patients’ clinical status or distance from the nodules cannot be avoided. Regarding the ALD samples, the cellular composition of the liver likely differ in the different patients with HCT occurring to different extents. An additional factor that may impact the ALD explants is the difference in the time of ischemia during the surgical procedure. Since access to ALD explants is limited, attempts to refine the classification of patients into groups to mitigate confounders is not possible. Overall, we agree with the reviewer’s remark, but we do feel that the pattern observed in the human liver samples is robust and consistent across the different approaches that were implemented (transcriptomics, western blotting, immunostaining).

**Reviewer #3**: This study aims to further understand the mechanisms controlling the transdifferentiation of hepatocytes into cholangiocytes (or so called HCT by the authors) which has been well studied in the DDC mouse model and more recently described in human liver affected by progressive MASLD. Analysing published data set, the authors confirm that HCT does not involve a intermediate progenitors state and they also uncover GRHL2 as a potential transcription factor regulating this process. These data are interesting and are in line with previous studies. The main interesting part is the role of GRHL2 and additional validations are necessary to validate the conclusions of this study.

1.	HCT has been widely studied in the DDC mouse model. These previous studies have already uncovered key epigenetic regulations and regulators such as Sox4 [ doi: 10.1038/s41467-024-45939-z.]. Similarly, a recent publication in human have demonstrated the existence of HCT in chronically injured liver and already established the absence of progenitor state [DOI: 10.1038/s41586-024-07465-2]. It would be very useful that the authors discussed their results in more details in the context of these previous publications.

As requested, we have extended the discussion to better position our findings in the context of the most recent literature. With regards to the 2 manuscripts mentioned by the reviewer (already cited in the original submitted version), we indicate that we corroborate the finding that SOX4 is induced during HCT and now also discuss its potential role within a network that would both activate cholangiocyte genes and repress the hepatocyte program (pages 18-19; see also the response to the question related to Figures 4-5 hereafter). The finding that HCT occurs in the absence of a progenitor-like state in human MASLD is now more clearly stated in a paragraph entirely dedicated to this subject where we indicate that our study strongly reinforces this model since our conclusions were drawn from cells with a proven hepatocyte origin thanks to lineage tracing in the mouse liver (pages 17-18).

In addition, we have included 2 additional studies published during the revision period of our study (PMID40169232 and PMID40281306). In PMID40169232, the Stanger group mined epigenetic regulators of HCT and identified a role for enzymes controlling H3K36me3 (NSD1 and KDM2A). In this study, EZH and H3K27me3 were not identified as modulators of HCT. However, *Ezh1* and *Ezh2* were silenced individually, which most probably explains these results since these 2 enzymes are redundant in hepatocytes and combined deletion of the 2 genes is required to trigger loss of H3K27me3 (PMID25477280). With regards to PMID40281306, this study used spatial transcriptomics to indicate that HCT occurs at the scar interface where an induction of *Grhl2* and *Esrp1* expression was found, which is fully in line with our study. These points have been added to the revised discussion pages 16-19.

2.	The epigenetic analyses (ChIP-Seq) are performed on very heterogeneous population. The hepatocytes represent 80% of the liver mass. However, they are extremely heterogenous due to zonation. This heterogeneity could play a key role in regenerative capacity and thus in the interpretation of their analyses.

As we indicate page 10, the chromatin obtained from mouse livers is mostly issued from hepatocytes, which is by far the largest population of cells and which is, moreover, mostly polyploid (up to 85% in C57BL/6 mice with mainly tetraploid hepatocytes) (PMID20861837). Moreover, we also used epigenome profiles obtained from isolated hepatocytes and not the whole liver including H3K27ac and H3K27me3 (Supplementary Table 4). In the revised manuscript, we have additionally mined published single-nuclei ATAC-seq data from mouse livers (PMID38182825). Using these data, we corroborate the differential chromatin status of genes from the C1-C3 clusters reported in Fig.3. Moreover, by comparing ATAC-seq signals in periportal *versus* pericentral hepatocytes, we show that zonation does not influence the chromatin accessibility of HCT genes (Supplementary Fig.4B; page 10). In sum, the data indicate that the results of our epigenetic analyses are without strong confounding bias.

However, we believe that a specific micro-environment promotes HCT. Indeed, in line with PMID40281306, our immunostaining data in human cirrhotic livers indicate that HCT occurs in hepatocytes found at the scar interface (Fig.8 and Supplementary Fig.11). This is now mentioned in the revised and extended discussion of our manuscript on page 17.

3.	The characterization of GRHL2 function is too limited and not convincing. Indeed, the data presented show that GRHL2 manipulation induces an epithelial signature but not a cholangiocytes signature. Indeed, they don't show the expression of cholangiocytes markers especially KRT19, KRT7, BICC1 etc. They need to provide data (QPCR and immunostaining with primary cells control cholangiocytes and hepatocytes) demonstrating that GRHL2 truly induces a change in cellular identity.

In the revised manuscript, we more clearly indicate that the Epith-Signature captures most cholangiocyte-enriched genes (pages 7-9). Indeed, Fig2B-E now show that induction of the Epith-signature genes is a primary event in HCT and includes archetypal cholangiocyte markers as highlighted in Fig.2E. Overall, more than 60% of the genes with enriched expression in cholangiocytes (when compared to other liver cell types), including commonly used cholangiocyte markers, are comprised within the larger Epith-Signature gene set (all gene lists are provided in Supplementary Table 2). In the revised manuscript, we highlight additional GRHL2 targets at the intersection between the Chol-enriched and Epith-Signature gene sets. This includes *ESRP1* and the cholangiocyte marker *KRT7* (Fig.6 and 7). This was moreover observed upon ectopic overexpression of GRHL2 *in vivo* in mouse hepatocytes (Fig.7). Direct regulation is further supported by GRHL2 binding to these genes (Fig.6G). In human liver samples with alcoholic hepatitis, co-labelling of GRHL2 and KRT7 (together with ALB) was evidenced (Fig.8E and Supplementary Fig.9D-E)

Altogether, these data indicate that GRHL2 induces the expression of commonly recognized cholangiocyte-enriched genes as part of a larger transcriptional program characterizing archetypal monopolarized epithelial cells. This is now further discussed on pages 18-19.

4.	HEPG2 or other transformed cells are a very poor model for these experiments. Tumor cells are likely impossible to transdifferentiate. They should use cholangiocytes organoids.

As requested by the reviewer, we have conducted additional experiments in which GRHL2 expression was induced in human organoids issued from EPCAM-positive cells grown in three-dimensional Matrigel and induced to differentiate according to PMID27560176 (characterization of this model, including expression of hepatocyte and cholangiocyte markers, is described in a manuscript in press at *JHEP Rep* – https://www.sciencedirect.com/science/article/pii/S2589555925002629). The data show a stimulation of GRHL2-target genes in this model together with decreased expression of hepatocyte marker genes (Fig.6J; page 14). The albeit limited induction of GRHL2 target genes we observed may be due to inherent limitations of this model including cellular heterogeneity, the need to use complex culture media with compounds that could impact on GRHL2 activities and the difficulty to achieve highly efficient viral transduction in matrix-embedded 3D organoid cultures. Importantly, results from additional experiments, in which GRHL2 was expressed in mouse hepatocytes *in vivo* using AAV8 vectors, revealed a more robust induction of GRHL2 target genes linked to a pronounced alteration of the hepatic parenchyma structure (Fig.7 and Supplementary Fig.8). GRHL2 also promoted the acquisition of a cyst-like organization by BMEL cells grown in 3D, reminiscent of in vivo cholangiocyte and epithelial cell behaviour (Fig.5G-H). Collectively, the wealth of experimental models used in our study robustly attest for the role of GRHL2 in the induction of epithelial genes contributing to acquisition of a cholangiocyte-like identity.

5.	Finally, the human data need to be reinforced.  They need to show that GRHL2 is expressed in biphenotypic cells co expressing KRT19/ALB for example.

As requested, we have performed co-immunostaining for ALB, KRT7 and GRHL2 in human liver failure samples. We used KRT7 as the cholangiocyte marker since it is directly induced by GRHL2 (Fig.6). We found triple-positive cells near scar tissue in line with the occurrence of HCT in this specific micro-environment (Fig.8E and Supplementary Fig.9C-D-E). Additional further mining of snRNA-seq data from MASH patient livers identified GRHL2-positive hepatocytes with mixed expression of hepatocyte and cholangiocyte identity genes (Supplementary Fig.10E). Altogether, these new data confirm that GRHL2 is expressed in biphenotypic cells expressing both hepatocyte and cholangiocyte markers.

Minor comments:

Figure 1F:

*       ALB expression should be investigated and shown to support the findings.

*Alb* expression was monitored and shows a pattern similar to that of Apoc3, further supporting our findings. Alb expression is shown in Supplementary Fig.1I.

*       Clarification is needed regarding the type of normalization used. Was normalization performed relative to a control group or endogenous gene expression?

Normalized expression data shown in Fig.1F are issued from our single-cell RNAseq data processing. Feature counts (i.e. genes counts) were normalized with the “LogNormalize“ function of Seurat. For each cell, gene counts were divided by the total number of counts, multiplied by a scale factor set to 10000 and then log-transformed. Gene expression was finally batch corrected. The Materials and Methods section was revised to provide a more detailed description of this data analysis procedure (pages 35-36).

*       It would be helpful to indicate the drawn trajectory on the UMAP visualization. Specifically, does the trajectory begin from cluster 4 and cross cluster 1? Is the trajectory determined only by the cells that overlap with cluster 1?

The trajectory for hepatocytes undergoing HCT as defined using pseudotime analyses is now shown in Fig.1E. This shows that the trajectory initiates at healthy hepatocytes and then link hepatocytes from cluster 3 (mostly issued from the liver of DDC-treated mice as shown in Fig.1C) to cholangiocytes.

Figure 2

*       Have cholangiocytes been traced to confirm they do not transdifferentiate into hepatocytes in this model?

To our knowledge, cholangiocytes have not been traced to monitor their transdifferentiation to hepatocytes in this model. DDC triggers bile duct obstruction leading to a cholestatic liver injury and, as such, the main regenerative event is expected to involve hepatocyte-to-cholangiocyte transdifferentiation (PMID32971004 and PMID31016659). This is now indicated on page 6.

Figure 4

*       GRHL2 Overexpression (OE): It should be demonstrated whether GRHL2 OE has any negative effects on hepatocyte differentiation. Additional data on the impact of GRHL2 ectopic expression on hepatocyte signature should be included.

*       BMEL Cells: Are genes involved in hepatocyte differentiation downregulated in BMEL cells in concert with GRHL2 OE? This aspect should be investigated and presented.

Figure 5

*       Similar to Figure 4, it is necessary to determine and present whether GRHL2 OE affects hepatocyte differentiation and signature-related genes.

In order to answer these points, we monitored the effect of GRHL2 expression on levels of hepatocyte-enriched genes used as an indicator of hepatocyte identity. Overall, we found that GRHL2 did not decrease the expression of these genes in HepG2 and BMEL cells (Supplementary Fig.7E). This may relate to the reviewer’s point on the limited plasticity of cell lines such as HepG2 cells. In line with this, the expression of hepatocyte markers was reduced upon GRHL2 ectopic expression in human organoids and in mouse hepatocytes *in vivo* (Fig.6 and 7). GRHL2 may therefore contribute to repression of the hepatocyte transcriptional program during HCT in addition to SOX4 (PMID 38409161) as indicated in the revised discussion pages 18-19.
