# Supplementary material for: Derepression of the epithelial transcription factor GRHL2 promotes direct hepatocyte-to-cholangiocyte transdifferentiation
Source: PLoS Biol. 2025 Dec 12;23(12):e3003547. doi: 10.1371/journal.pbio.3003547 (PMC12714216; doi:10.1371/journal.pbio.3003547)
Supplement: Attachment — Submitted filename: 2025_17_11_Rebuttal.docx [file pbio.3003547.s031.docx]

**Response to reviewers:**

Reviewer #1: The revised manuscript by Vasseur et al. addresses the points raised in the initial review. The revised manuscript convincingly shows that hepatocyte-to-cholangiocyte transdifferentiation can be driven by derepression of GRHL2 and activation of a canonical epithelial transcriptional program, rather than by reactivating a progenitor-like state. The revised manuscript includes substantial new data that strengthen the mechanistic and functional analyses. The revised manuscript is clearly written, well contextualized, and ready for publication.

Response to previous concerns

1. Definition of the cholangiocyte-like transcriptional program: The authors now define this program as induction of an "Epith-Signature" capturing over 60% of cholangiocyte-enriched genes. They included complete gene lists (Suppl. Table 2), full sets of GRHL2-induced genes (Suppl. Tables 6 and 8), and highlighted direct targets such as ESRP1 and KRT7 (Figs. 6, 7). The role of SOX4 is clarified (p. 18).

2. Clarification of EZH2 effects in the discussion: The confusing statement regarding Yang et al. (2023) has been corrected to state that EZH2 inhibition promotes cholangiocyte differentiation, which is consistent with the authors' findings.

3. Addition of mechanistic and functional evidence: New data show direct binding of EZH2 and H3K27me3 at the Grhl2 promoter (Fig. 5), derepression of Grhl2 after Ezh1/2 loss, and activation of Epith-Signature genes. GRHL2 overexpression promotes 3D cyst formation in BMEL cells and reprogramming of hepatocytes in vivo (Figs. 5-7).

4. Integration with recent literature and human data: The revised discussion references recent studies (SOX4/H3K36me axis, MASLD, spatial transcriptomics) and emphasizes concordance of findings in mice with human alcoholic hepatitis, PSC, and PBC samples showing GRHL2+/KRT7+ hepatocytes at scar regions (Fig. 8).

5. Additional experimental validation: The authors performed new GRHL2 expression experiments in human liver organoids, confirming the induction of epithelial/cholangiocyte genes and repression of hepatocyte markers (Fig. 6J). Zonation and cell-type heterogeneity were examined using snATAC-seq and hepatocyte-specific epigenomic data.

We would like to thank reviewer #1 for the insightful comments which helped improved our study and for the positive feedback on our revised manuscript.

Reviewer #2, Pinglong Xu (note, Reviewer 2 has signed this review): The questions raised during my initial review have largely been addressed and the manuscript is significantly improved.

We would like to thank reviewer #2 for the insightful comments which helped improved our study and for the positive feedback on our revised manuscript.

Reviewer #3: The authors have done a lot of efforts to answer the reviewers' comments. The data showing that GRHL2 plays a role in the transdifferentiation of hepatocytes into cholangiocytes are indeed convincing. However, the problem remains the capacity of GRHL2 to directly drive the transdifferentiation of hepatocyte into cholangiocytes. The functional data either in vitro or in vivo show that GRHL2 overexpression results in the up regulation of epithelial markers which are expressed in a number of cells (EHF, CLDN4). KRT7 and ESPR1 are indeed specific for cholangiocytes. However, KRT7 is associated with functional maturation and KRT19 is more broadly used. What about other specific/functional markers markers which are universally used such as KRT19, HNF1B, CFTR, BICC1, EPCAM, DCD2 etc These markers are commonly used to characterise cholangiocytes. Why are they not using these markers in Figure 5, 6 and 7. It seems that the authors have handpicked few markers which can support their conclusions. Of note the data in human mainly show that GRHL2 is expressed in ductular reaction. These cholangiocytes are usually located closed to the regenerative nodule but this does not mean that they are produced by hepatocytes. Furthermore, only few biphenotypic cells truly express GRHL2. So again GRHL2 is likely to participate in the transdifferentiation process but not to drive it. This factor is not sufficient for this process. So, it would be incredibly useful either to include the markers mentioned above in Figure 6 and 7 or to adapt their conclusions/title to reflect their results more accurately.

In line with the reviewer’s point, while we believe our study convincingly demonstrates a role for GRHL2 in HCT, our aim was not to convey that GRHL2 alone is sufficient to induce this transdifferentiation. For instance, our Discussion already clearly states that additional regulatory pathways and transcriptional regulators are required to induce cholangiocyte genes such as CFTR (page 18). To make this point even clearer, we have added the following sentence on page 18: “GRHL2 induced expression of some classical cholangiocyte markers (e.g., *KRT7*), but not all commonly used ones, indicating that GRHL2 is most likely not sufficient to drive full HCT.” We have also modified the title and abstract to further tone down our main conclusion stating that GRHL2 “promotes” (rather than “drives”) HCT.

**Editorial points:**

1) ETHICS STATEMENT: Please update your ethics statement to include the approval number for the protocol approved by the Lille Ethical Committee.

The approval numbers have been added.

2) DATA: Thank you for providing the CHIP-Seq and RNA-seq data on GEO. Please add a note to every relevant figure legend, pointing readers to these datasets. (ex you can say "the data underlying this figure can be found at ___")

The following sentence has been added to the legend of all relevant primary and supplementary figures: “The original data underlying this figure can be found at the Gene Expression Omnibus (GSE281717)”.

3) DATA: In addition to the data you have already provided, we also need you to provide the underlying data for all other figures in your paper. While the RNA-seq and CHIP-seq data are fine, we also need the underlying data for your qPCR and Western blot analysis, etc (ex Fig 8A-B)

Note that we do not require all raw data. Rather, we ask that all individual quantitative observations that underlie the data summarized in the figures and results of your paper be made available in one of the following forms:

The requested additional raw data are now provided in the the S1_data.xls file.

a. Supplementary files (e.g., excel). Please ensure that all data files are uploaded as 'Supporting Information' and are invariably referred to (in the manuscript, figure legends, and the Description field when uploading your files) using the following format verbatim: S1 Data, S2 Data, etc. Multiple panels of a single or even several figures can be included as multiple sheets in one excel file that is saved using exactly the following convention: S1_Data.xlsx (using an underscore).

A sentence indicating that the numerical values of individual biological replicates or mouse livers or human samples is provided in the S1 data file has been added to all relevant primary and supplementary figures.

b. Deposition in a publicly available repository. Please also provide the accession code or a reviewer link so that we may view your data before publication.

All ChIP-seq and RNA-seq data generated in this study have been deposited into the Gene Expression Omnibus SuperSeries under accession number GSE281717. Reviewer access is provided by the following token: ajkrueiknlwllgj.

NOTE: the numerical data provided should include all replicates AND the way in which the plotted mean and errors were derived (it should not present only the mean/average values).

Please also ensure that figure legends in your manuscript include information on where the underlying data can be found, and ensure your supplemental data file/s has a legend.

Please ensure that your Data Statement in the submission system accurately describes where your data can be found.

This has been verified.

4) CODE: Thank you for depositing the code for your study on Zenodo. I see that those files are currently set to private, and I could not access the code (even when I used the reviewer links that you provided). Can you provide me with reviewer access so that I can verify that the code deposition meets our requirements? Note that you will need to make all code and data public before publication.

Codes and data deposited in Zenodo have now been made publicly available.

Data: https://zenodo.org/records/13897729

Code: https://zenodo.org/records/13897656

Those links have been updated in the manuscript.

5) BLOT AND GEL IMAGES: Thank you for providing a file with the raw western blot images for your study. The images that you provided look a bit cropped, and so I ask that you edit this file to include the fully uncropped images taken from the scanner.

All uncropped images are now shown. Some western blot images (Fig. 8B – H3 and H3K27me3) were obtained from membranes that had been cut prior to antibody incubation, in order to assess proteins of higher molecular weight in parallel. This approach was applied only after confirming the specificity of the antibodies and the absence of non-specific bands. The raw images have been extended to clearly show the entire membrane section used in each case.

**Technical requests:**

We do not publish any copyright or trademark symbols that usually accompany proprietary names, eg  ,  , ™  (e.g. next to drug or reagent names). Please remove all instances of trademark/copyright symbols throughout the text, including:

  on pages 21, 22, 23, 26, 27, and 28.

™ on page 30.

Trademark/copyright symbols have been removed.
